# The halophilic bacteria *Gracilibacillus dipsosauri* GDHT17 alleviates salt stress on perennial ryegrass seedlings

**DOI:** 10.3389/fmicb.2023.1213884

**Published:** 2023-07-26

**Authors:** Xiangying Li, Jinyuan Zheng, Wei Wei, Zifan Gong, Zhenyu Liu

**Affiliations:** College of Plant Protection, Shandong Agricultural University, Tai’an, Shandong, China

**Keywords:** salt stress, *Gracilibacillus dipsosauri* GDHT17, saline habitats, ryegrass, halotolerant bacteria

## Abstract

**Introduction:**

Adverse abiotic environmental conditions including excess salt in the soil, constantly challenge plants and disrupt the function of plants, even inflict damage on plants. Salt stress is one of the major limiting factors for agricultural productivity and severe restrictions on plant growth. One of the critical ways to improve plant salt tolerance is halotolerant bacteria application. However, few such halotolerant bacteria were known and should be explored furtherly.

**Methods:**

Halophilic bacterium strain was isolated from saline soil with serial dilution and identified with classical bacteriological tests and 16S rRNA analysis. Perennial ryegrass (*Lolium perenne* L) was used in this study to evaluate the potential effect of the bacteria.

**Results and discussion:**

A halophilic bacterium strain GDHT17, was isolated from saline soil, which grows in the salinities media with 1.0%, 5.0%, and 10.0% (w/v) NaCl, and identified as *Gracilibacillus dipsosauri*. Inoculating GDHT17 can significantly promote ryegrass’s seedling height and stem diameter and increase the root length, diameter, and surface area at different salt concentrations, indicating the significant salt stress alleviating effect of GDHT17 on the growth of ryegrass. The alleviating effect on roots growth showed more effective, especially on the root length, which increased significantly by 26.39%, 42.59%, and 98.73% at salt stress of 100 mM, 200 mM, and 300 mM NaCl when the seedlings were inoculated with GDHT17. Inoculating GDHT17 also increases perennial ryegrass biomass, water content, chlorophyll and carotenoid content under salt stress. The contents of proline and malonaldehyde in the seedlings inoculated with GDHT17 increased by 83.50% and 6.87%, when treated with 300 mM NaCl; however, the contents of MDA and Pro did not show an apparent effect under salt stress of 100 mM or 200 mM NaCl. GDHT17-inoculating maintained the Na^+^/K^+^ ratio in the salt-stressed ryegrass. The Na^+^/K^+^ ratio decreased by 26.52%, 6.89%, and 29.92% in the GDHT17-inoculated seedling roots treated with 100 mM, 200 mM, and 300 mM NaCl, respectively. The GDHT17-inoculating increased the POD and SOD activity of ryegrass seedlings by 25.83% and 250.79%, respectively, at a salt stress of 300 mM NaCl, indicating the properties of GDHT17, improving the activity of antioxidant enzymes of ryegrass at the salt-stress condition. Our results suggest that *G. dipsosauri* GDHT17 may alleviate salt stress on ryegrass in multiple ways; hence it can be processed into microbial inoculants to increase salt tolerance of ryegrass, as well as other plants in saline soil.

## 1. Introduction

Salt stress is a severe abiotic factor that reduces yields and physiological processes in many plants worldwide, including crops, vegetables, and trees (Ghosh and Ali Md, 2016; Tomaz et al., 2020; Zhang et al., 2022). It has been estimated that about 7.5% of land in the world is severely affected by soil salt stress (Li et al., 2019; Hassani et al., 2020). Moreover, climate change, poor agricultural practices, and industrial pollution cause a 10% annual rise in global soil salinization (Ziska et al., 2012; Zhang et al., 2016). Soil salt stress has become crucial to agricultural productivity and sustainable development. In plants, the adverse effects of salt stress usually cause physiological drought by high solute concentrations leading to water deficit limiting plant water use in the soil, as well as ion toxicity caused by ion and osmolarity imbalances leading to oxidative stress (De Souza Miranda et al., 2017; Ullah et al., 2022). The impacts of salt stress on plant physiological and biochemical processes include nutrient uptake, osmotic stress balance, oxidative stress, photosynthetic rate, and overall growth, and ultimately reduced crop yield (Ji et al., 2018; Chang et al., 2019; Petretto et al., 2019).

To counteract the adverse effects of salt stress, plants adopt a number of fundamental mechanisms, including exclusion of toxic ions (Na^+^ and Cl ^-^), synthesis of endogenous metabolites associated with stress adaptation, e.g., proline, betaine, etc., synthesis and activation of antioxidant enzymes such as catalase (CAT), superoxide dismutase (SOD), peroxidase (POD), etc., and non-enzymatic antioxidants such as carotenoids, phenolic compounds etc. (Rahman et al., 2019; Zaid and Wani, 2019; Patel et al., 2020; Mostofa et al., 2021; Zhang et al., 2022). The salt tolerance mechanism of plants can be used as a standard for evaluating plant salt damage. In addition, it is meaningful and urgent to improve the salt tolerance of plants and alleviate the damage to plants caused by salt stress. Therefore, it is imperative to use appropriate technologies to alleviate salt stress damage to crops.

Advanced and more effective technologies for improving crop yields in saline soils should be explored. Genetic factors are essential in determining salt tolerance of plants, and some genes were employed for genetic modification to improve salt tolerance in plants (He et al., 2019; Zhang et al., 2022). However, access to salt-tolerant varieties is rare and requires considerable time and financial resources. Fortunately, microbial inoculation has been proposed to improve salt tolerance in crops and has received extensive attention (Li et al., 2016; Kearl et al., 2019). Some microorganisms, including *Rhizobium*, *Bacillus*, *Pseudomonas*, *Pantotrichum*, *Burkholderia*, *Microbacterium*, *Methylobacterium*, *Variovorax*, *Enterobacter*, etc., have been demonstrated with the properties of improving plant tolerance under different abiotic stresses (Yildirim et al., 2008; Dey et al., 2021; Shahid et al., 2022). Therefore, exploiting and employing such microorganisms for plant salt tolerance is a promising alternative. The mechanisms enhancing salt tolerance in plants of these microorganisms are mostly similar in different taxa (Saum and Müller, 2007; Albacete et al., 2008; Egamberdieva et al., 2016). These bacteria mediate salt tolerance in plants by regulating the expression of reactive oxygen species (ROS) scavenging enzymes (Khodair et al., 2008; Poli et al., 2010), altering the selectivity of Na^+^, K^+^, and maintaining high K^+^/Na^+^ ratios in plants (Ramezani et al., 2011; Rojas-Tapias et al., 2012; Pinedo et al., 2015). For example, *Kosakonia radicincitans* strain KR-17 applied to salt-stressed plants significantly reduced plant membrane damage, stress metabolites, and antioxidant defense enzymes (Shahid et al., 2022). Orhan et al. showed that inoculation of halotolerant and halophilic bacteria attenuated NaCl-induced toxicity and increased wheat yield in saline soils (Orhan, 2016). However, the bacteria of improving salt tolerance in plants led by microorganisms have yet to be elaborate.

Natural saline habitats are a resource bank of excellent salt-tolerant bacteria. Many salt-tolerant microorganisms have been found in natural saline environments (Hovik et al., 2018), and these microorganisms are most suitable for growth in salt-stressed environments. For example, Priestia aryabhattai JL-5 derived from saline soil has been used to promote the saline-alkaline tolerance of *Leymus chinensis* (Wang et al., 2023). Perennial ryegrass (*Lolium perenne* L.) is widely planted as a cold-season turf grass and a crucial forage grass, with a high yield and strong regeneration (Wu et al., 2005; Kane, 2011). Perennial ryegrass has strong tillering and regeneration abilities (Wang and Bughrara, 2009; Kane, 2011); however, the salt tolerance in commercial cultivars is only moderate, and the growth of ryegrass is usually affected by salt stress.

In the study, we isolated salt-tolerant bacteria from saline habitats in the Yellow River Delta, where severe land salinization is marked. We employed perennial ryegrass to evaluate the effect of the bacteria on the plant under salt stress, expecting to explore the potential properties of the bacteria to alleviate the salt stress on plants.

## 2. Materials and methods

### 2.1. Bacteria isolation and identification

The selinene soil samples with a pH of 8.5 were collected in July from the Yellow River Delta (37° 22 ‘N-38° 04 ‘N, 118° 14 ‘E-119° 05 ‘E), located in Shandong Province, China. Samples were packed in ice, brought to the lab, and stored at 4°C for processing. As previously described (Suman et al., 2018), LB solid medium (10 g Tryptone, 5 g Yeast, 15–18 g agar, 1 L distilled water) with NaCl concentrations of 0.5, 1, 5, 10, and 15% were prepared for bacteria isolation. Soil samples were serially diluted with ten-fold dilutions up to 10^−5^ in sterile water. 100 μL of soil diluent were plated on the solid LB with different NaCl concentrations and incubated at 28°C for 48 h to 72 h. The colonies, which grow on solid LB with 10 and 15% NaCl with distinct bacterial colony morphology, were selected, purified, and cultured in LB with 5% NaCl (w/v) at 28°C and then preserved at 4°C for further testing.

Cultural, morphological, and physiological characteristics of the bacteria were carried out using conventional methods (Carrasco et al., 2006; Jeon et al., 2008). The colony morphology was observed after culture on solid LB media with 5% NaCl (w/v) at 28°C for 48 h. The characters of gram staining and spore staining were observed with OLYMPUS-BX51 optical microscope.

The biochemical characteristics of bacteria were determined after culture in LB media with 5% NaCl (w/v) at 28°C for 48 h. Catalase activity, oxidase activity, Voges-Proskauer test, gelatin hydrolysis, and starch degradation tests were determined as described previously (PhilippGerhardt, 1981; Ventosa et al., 1982; Chen et al., 2007).

For 16S rRNA identification, the bacteria was cultured in LB media with 5% NaCl (w/v) at 28°C for 48 h. Genomic DNA was extracted using an OMEGA bacterial DNA kit according to the manufacturer’s instructions. The primers, forward (5′-AGAGTTTGATCCTGGCTCAG-3′) and Reverse (5′-AAGGAGGTGATCCAGCCGCA-3′) were used for the 16S rRNA gene amplification. The sequence of 16S rRNA was analyzed by comparing it with the 16S rRNA genes available at the GenBank database of the National Center for Biological Information (NCBI) using the BLAST algorithm. Phylogenetic analysis of 16S rRNA was performed using the MEGA version 11 (Tamura et al., 2021).

### 2.2. Determination salt tolerance properties of bacteria

The media with 1, 5, 10, and 15% NaCl concentration were used for salt tolerance properties determination by determining the culture properties. The selected strain was cultured in LB with 5% NaCl (w/v) at 28°C for 24 h. The bacteria culture was then streaked on the solid LB media with NaCl concentrations of 1, 5, 10, and 15%. Furthermore, the bacteria were inoculated in LB with 1, 5, 10, and 15% NaCl concentration and shaking cultured at 180 r · min^−1^, 28°C. The OD value at 600 nm of the bacterial solution was measured every 12 h.

### 2.3. Plant growth conditions and plant inoculation

The seeds of perennial ryegrass (*L.perenne* L. cv. *Taya*) were surface sterilized and rinsed with sterile deionized water. The Hogland nutrient solution with half concentration (5 mM KNO_3_, 1 mM NH_4_H_2_PO_4_, 0.5 mM Ca (NO_3_)_2_, 0.5 mM MgSO_4_, 92 μM H_3_BO_3_, 60 μM Fe-citrate, 1.6 μM ZnSO_4_·7H_2_O, 0.6 μM CuSO_4_·5H_2_O, 18 μM MnCl_2_·4H_2_O, and 0.7 μM (NH_4_)_6_Mo_7_O_24_·4H_2_O) (Chen et al., 2022) was prepared. The culture solution was obtained by adding NaCl into half concentration Hogland nutrient solution; then, the surface sterilized seeds were sown in the sterilized perlites floated on the culture solution with a salt concentration of 0 mM, 100 mM, 200 mM, and 300 mM. 100 μL bacteria cultured for 48 h were inoculated in the culture solution. Each treatment was set with 10 repetitions. The treatment were set in illuminating incubator and cultured at 25°C, 12 h/12 h for light/dar, supplying the culture solution periodically. 30 days after, 20 seedlings were collected for growth biomass determination and physiological and biochemical property determination.

### 2.4. Plant characters measurement

#### 2.4.1. Growth biomass measurement

The plant height was measured using the scale ruler with the minimum millimeter, and the stem diameter was measured with a vernier caliper. The total root length, diameter, and surface area were measured by the root scanner (WinRHIZO Plant Root Scanning System, Software name WinRHIZO).

For the relative water content test, the fresh weight (FW) of leaves and stems was weighed immediately after the sample collection, then the dry weight (DW) was measured after the samples dried at 80°C for 24 h. The water content (WC) was calculated as follows:


WC(%)=(FW−DW)/FW×100

#### 2.4.2. Photosynthetic pigments determination

The chlorophyll and carotenoid were measured with fresh leaves, following the protocol described by the classical methodology laboratory (Zhao et al., 2020). Briefly, weigh 0.1 g of fresh leaves; the fresh leaf samples were completely bleached, extracted with 80% acetone, and then centrifuged at 13,000 rpm for 10 min to obtain the chlorophyll supernatants. The chlorophyll a, chlorophyll b, and carotenoid were determined at 663 nm, 645 nm, and 470 nm absorbance, respectively. Ryegrass seedling leaf chlorophylls and carotenoid contents were calculated as follows:


$$\begin{array}{l} Chlorophyll\;a\;content\;\left(mg/g\right)=\left(12.7\times A_{663}-2.59\times A_{645}\right)\\ \times 10÷1000÷0.1; \end{array}$$

$$\begin{array}{l} Chlorophyll\;b\;content\;\left(mg/g\right)=\left(22.9\times A_{645}-4.69\times A_{663}\right)\\ \times 10÷1000÷0.1; \end{array}$$

$$\begin{array}{l} Chlorophylltotalcontent\;\left(mg/g\right)\\ =\left(20.21\times A_{645}+8.02\times A_{663}\right)\times 10÷1000÷0.1; \end{array}$$

$$\begin{array}{l} Carotenoidcontent\;\left(mg/g\right)\\ =\left(1000\times A_{470}-3.31\times C_{a}-104\times C_{b}\right)÷229\times 10÷1000÷0.1. \end{array}$$

A_663_ represented the absorbance value at 663 nm; A_645_ represented the absorbance value at 645 nm; A_470_ represented the absorbance value at 470 nm; C_a_ represented the chlorophyll a content; C_b_ represented the chlorophyll b content.

#### 2.4.3. Malondialdehyde and proline assay

0.1 g ryegrass seedling leaves were harvested, immediately ground into powders with liquid nitrogen, and homogenized in 4 mL ice-cold phosphate buffer (50 mM, pH 7.8). The homogenates were then centrifuged at 12,000 rpm at 4°C for 20 min. The supernatant was collected and used to determine the malondialdehyde (MDA) content following the method described by Hu et al. using the thiobarbituric acid reaction method (Hu et al., 2011). Absorbance was determined at 532 (M_532_) and 600 (M_600_) nm to estimate MDA content. The malondialdehyde content was calculated as follows:


$$MDA\;\left(nmol/g\right)=32.258\times \left(M_{532}-M_{600}\right)÷0.1$$

The Proline (Pro) content of the leaves was determined following a standard protocol with slight modifications (Bates et al., 1973). Ryegrass seedling leaves were homogenized in 5% (w/v) sulfosalicylic acid and centrifuged at 8,500 g for 10 min. The resulting supernatant was added with 2% ninhydrin (w/v) and incubated at 100°C for 30 min. An equal volume of toluene was added to the mixture to obtain the upper aqueous phase. The absorbance was measured at 520 nm. A standard curve of pure L-proline was used for the proline content calibration.

#### 2.4.4. Ionic accumulation analysis

1 g of seedling leaves or roots from each treatment was selected for analysis of ionic elements. These samples of leaves or roots were rinsed in double distilled water. Roots and shoots were separated, and samples were ground in liquid nitrogen and then digested in 100 mL of a mixture of 10:1:2 perchloric acid, sulphuric acid, and distilled water at heating by an alcohol lamp. The Na^+^ and K^+^ content was measured using an atomic absorption spectrophotometer (AAS 2380, Perkin Elmer, United States). The Na^+^/K^+^ ratio was calculated afterward. Each treatment was performed in triplicate.

#### 2.4.5. Measurement of the enzyme activity of SOD, CAT, and POD

Antioxidant enzymes were extracted, and superoxide dismutase (SOD), catalase (CAT), and peroxidase (POD) activities of the leaves were measured according to the instructions of the kit (Solarbio, cat nos. BC0175, BC0205, BC0095). Briefly, 1 g of fresh ryegrass seedling leaves were harvested and ground in liquid nitrogen, and the enzymes were extracted using an extraction buffer. Subsequently, the extract containing the enzymes was centrifuged at 12,000 g for 10 min, and the supernatant was aspirated for the determination of enzyme activity. Each activity unit is defined according to the instructions of the antioxidant enzyme assay kit.

### 2.5. Statistical analysis

GraphPad Prism 8 software was used for data collation and graphing. One-way ANOVA (*p* < 0. 05) was performed using IBM SPSS statistics 20 software, and Duncan’s method was used for multiple comparisons. Marking of significant differences.

## 3. Results

### 3.1. Isolation and identification of halotolerant bacteria

In the bacteria isolated from the selinene soil samples, a few bacteria colonies grew on the solid LB with 10% or 15% NaCl, and many colonies grew on the solid LB with 1% NaCl or 5% NaCl; however, the number of colonies grew on the solid LB with 0.5% NaCl was less than that on the solid LB with 1% NaCl and more than that on the solid LB with 10% NaCl. We obtained 160 isolates in total from the saline soil samples, and in these isolates, 5 isolates grew on the solid LB with 15% NaCl, and 12 isolates grew on the solid LB with 10% NaCl. A strain that grew on the solid LB with 10% NaCl, was chosen for further study and named GDHT17.

**Figure 1:**
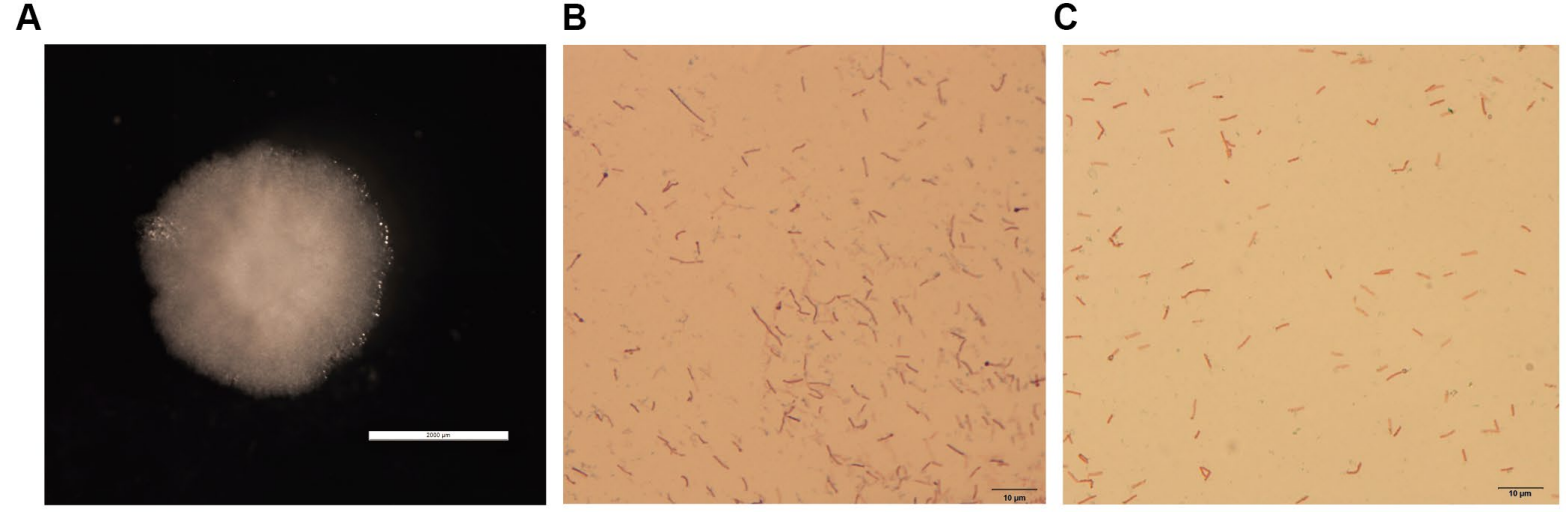
Isolation and identification of halotolerant bacteria. **(A)** Colony morphology of strain GDHT17 on LB solid medium, the scale of panel A is 2000 μm. **(B)** Gram stain, the scale of panel B is 10 μm. **(C)** Spore staining character, the scale of panel C is 10 μm.

The colonies of GDHT17 were circular, moist, smooth, raised, undulated, rough, opaque, and grayish-white with no diffusible pigment (Figure 1A). The bacteria consisted of motile, aerobic, Gram-positive, long rods (Figure 1B), showed terminal endospore formation (Figure 1C), and were positive for catalase activity, oxidase activity, Voges-Proskauer test, gelatin hydrolysis, and starch degradation.

**Figure 2:**
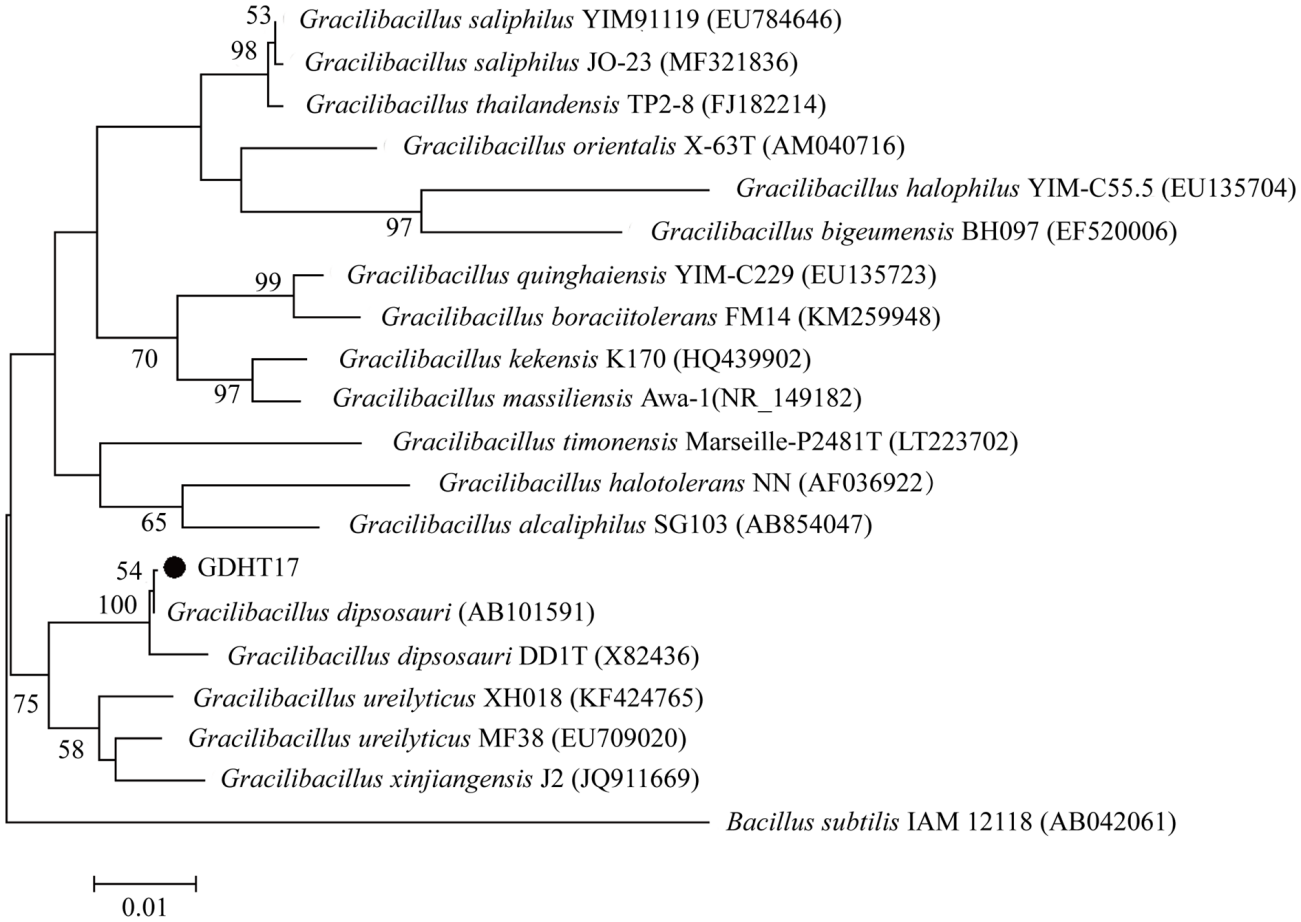
Phylogenetic trees based on 16S rRNA gene sequences using the maximum-likelihood methods, bar 0.01 substitutions per nucleotide position.

The 16S rRNA gene sequence of GDHT17 was obtained and deposited in GenBank with the accession number MG980060. The 16S rRNA gene sequence identities between strain GDHT17 and the type strains of other recognized members of the genus *Gracilibacillus* were below 97%. The sequence of GDHT17 shared 99.9% and 99.1 identities with those of *G. dipsosauri* (X82436) and *G. dipsosauri* DD1T (AB101591), respectively. Some typical 16S rRNA gene sequences belonging to *Gracilibacillus* and the 16S rRNA gene sequence of GDHT17 were employed for phylogenetic analysis (Figure 2), which showed higher similarities between GDHT17 and *G. dipsosauri*. According to the classical bacteriological tests and the 16S rRNA gene sequence analysis, we affirmed that the strain GDHT17 belongs to *G. dipsosauri*.

### 3.2. Salt tolerance properties of strain GDHT17

**Figure 3:**
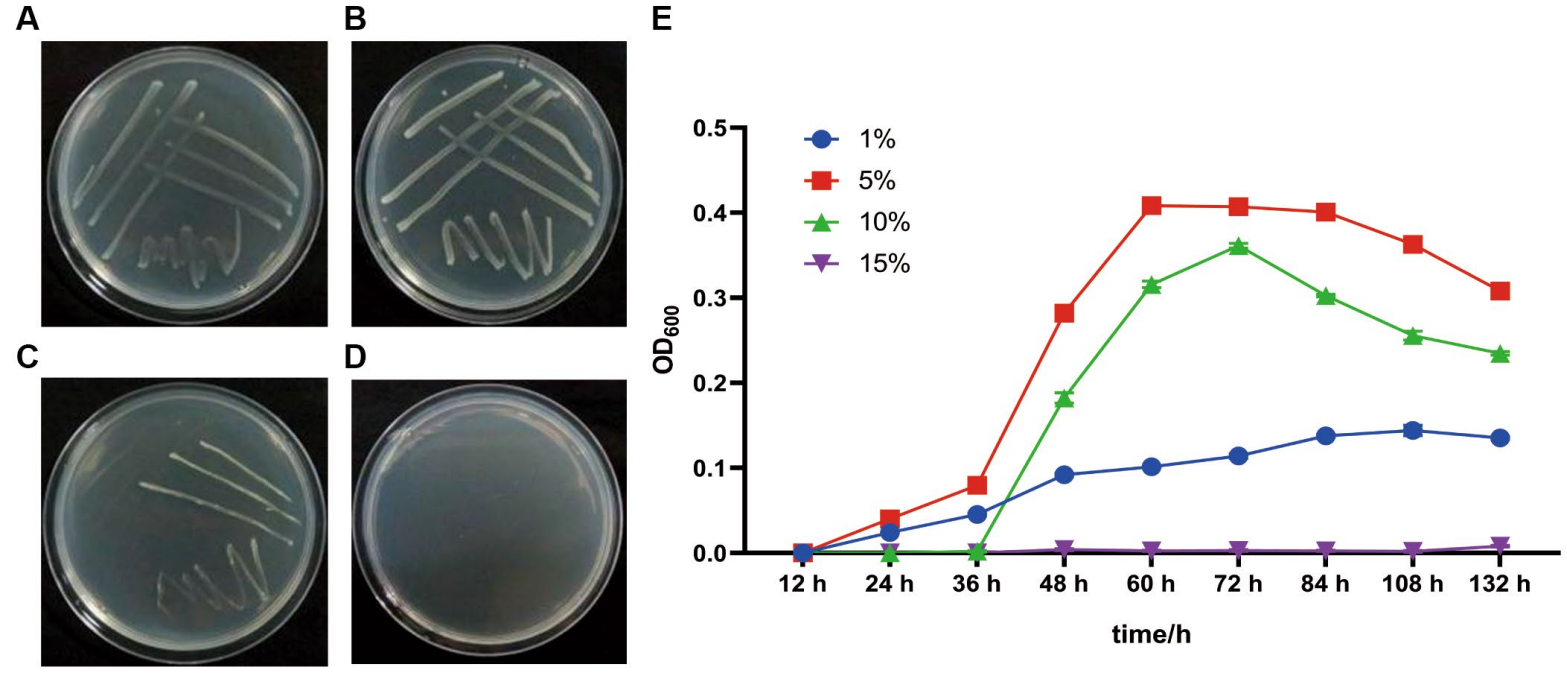
Salt tolerance properties of *G. dipsosauri* GDHT17. The patterns of GDHT17 on salt-containing solid LB plates cultivated for 48 h, **(A–D)**, the solid LB plates with 1.0, 5.0, 10.0, and 15.0% NaCl (w/v); **(E)** the growth curve of GDHT17 cultivated in LB containing with different salt concentrations. The line graph indicates the mean of the three replicates (*n* = 3). Error bars indicate the standard deviation (SD) of the three replicates.

*G. dipsosauri* GDHT17 grows on the LB plates and in LB broth with salinities of 1.0, 5.0, and 10.0% (w/v) NaCl; the optimum is 5.0% (Figure 3). However, it does not grow in the media with 15.0% NaCl (Figure 3). Therefore, *G. dipsosauri* GDHT17 is considered a moderately halophilic bacterium.

### 3.3. Inoculation of GDHT17 positively affected the growth and biomass of NaCl-treated ryegrass

**Figure 4:**
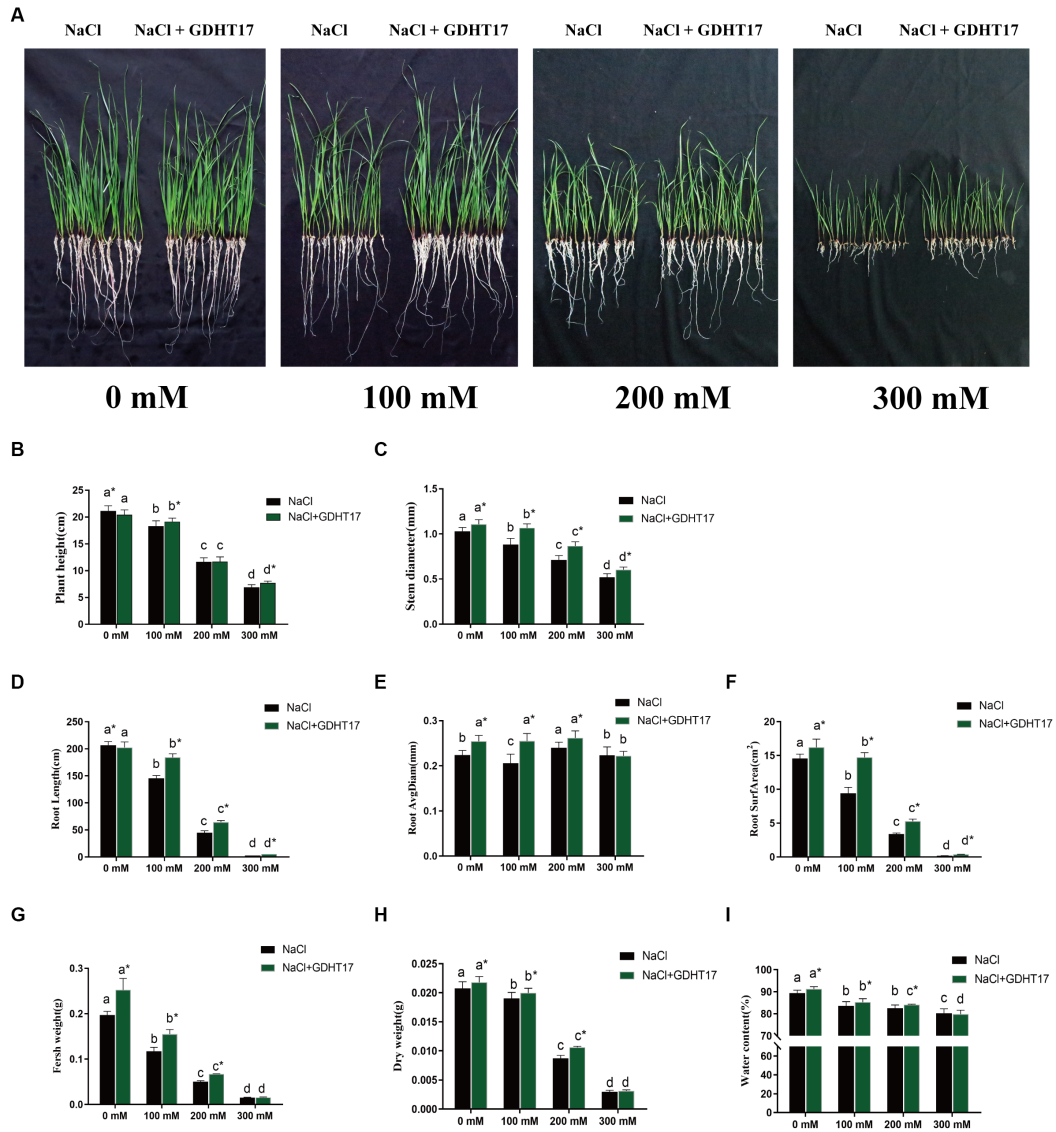
Inoculation of *G. dipsosauri* GDHT17 Affected Stressor. **(A)** Growth of ryegrass seedlings in non and inoculated with GDHT17 Hoagland nutrient solutions when treated with 0 mM, 100 mM, 200 mM, and 300 mM NaCl; Effect of inoculation with GDHT17 on the growth and biomass of ryegrass seedlings. **(B)** Seedling height, **(C)** stem diameter, **(D)** total root length, **(E)** root diameter, **(F)** root surface area, **(G)** fresh weight, **(H)** dry weight, **(I)** water content. Bar diagrams represent the mean of 20 replicates (*n* = 20). Error bars indicate the standard deviation (SD) of the 20 replicates. Different lowercase letters mean NaCl or NaCl+GDHT17; there were significant differences when different concentrations of NaCl treatments (*p* < 0.05). Bars diagrams marked with “*” indicate significant differences between NaCl and NaCl+GDHT17 treatment in the same NaCl concentration (*p* < 0.05).

Salt stress significantly inhibits the seedling height and stem diameter of ryegrass when treated with 100 mM, 200 mM, and 300 mM NaCl in the Half concentration of Hogland nutrient solution (Figures 4A–C). The seedling height and stem diameter significantly decreased with the salt concentration increase. The same happened when GDHT17 was inoculated in the Hogland nutrient solution. However, inoculating GDHT17 significantly promote the ryegrass’s seedling height and stem diameter at different salt concentrations, except for 200 mM NaCl (Figures 4A,B). Compared with the non-bacteria inoculated seedlings, GDHT17 significantly increased the seedling height by 4.59 and 12.16% at 100 mM and 300 mM NaCl stress, respectively (Figures 4A,B), significantly increased the stem diameter by 20.98, 21.75 and 15.98% at 100 mM, 200 mM, and 300 mM NaCl stress, respectively (Figures 4A,C). Therefore, GDHT17 alleviates the effects of salt stress on the growth of seedling height and stem diameter of ryegrass.

The root length and root surface area of the seedlings were inhibited when treated with different concentrations of NaCl; the root length and root surface area significantly decreased with the salt concentration increase. However, when treated with different concentrations of NaCl, the seedling root diameter did not decrease with increasing salt concentration (Figures 4D,E); only at the concentration of 300 nM NaCl, the root diameter was significantly inhibited (Figure 4F). The root length, diameter, and surface area of ryegrass seedlings increased when the GDHT17 were inoculated.

The root length increased significantly by 26.39, 42.59, and 98.73% at salt stress of 100 mM, 200 mM, and 300 mM NaCl, respectively, compared with the non-GDHT17 inoculation treatment (Figure 4D). The root diameter significantly increased by 13.60% in non-salt conditions, 23.76, and 6.16% at salt stress of 100 mM and 200 mM NaCl, respectively, compared with the non-GDHT17 inoculation treatment. However, GDHT17 had no significant increased effect on the root diameter under 300 mM NaCl stress (Figure 4E). The root surface area significantly increased by 11.29% in non-salt conditions, and the root surface area significantly increased by 56.30, 55.96, and 97.73% at salt stress of 100 Mm, 200 mM, and 300 mM NaCl, respectively, compared with the non-GDHT17 inoculation treatment (Figure 4F). In conclusion, GDHT17 can alleviate the salt stress on the root growth of ryegrass. Indeed, in the case of 300 mM NaCl treatment, the root length and surface were significantly decreased, demonstrating severe damage to the ryegrass even if the inoculation of GDHT17.

The seedling biomass and water content decreased when treated with different concentrations of NaCl (Figures 4G–I). Inoculating GDHT17 can significantly increase ryegrass’s seedling biomass at different salt concentrations, except for 300 mM NaCl (Figures 4G–I). The fresh weight, dry weight, and water content significantly increased by 27.80, 4.70, and 2.05%, respectively, in non-salt conditions, compared with the non-GDHT17 inoculation treatment. In summary, GDHT17 can increase the biomass and water content of ryegrass, alleviating the inhibitory effect of salt stress on the growth of ryegrass.

### 3.4. Inoculation of GDHT17 positively affected the chlorophyll and carotenoid content of NaCl-treated ryegrass

**Figure 5:**
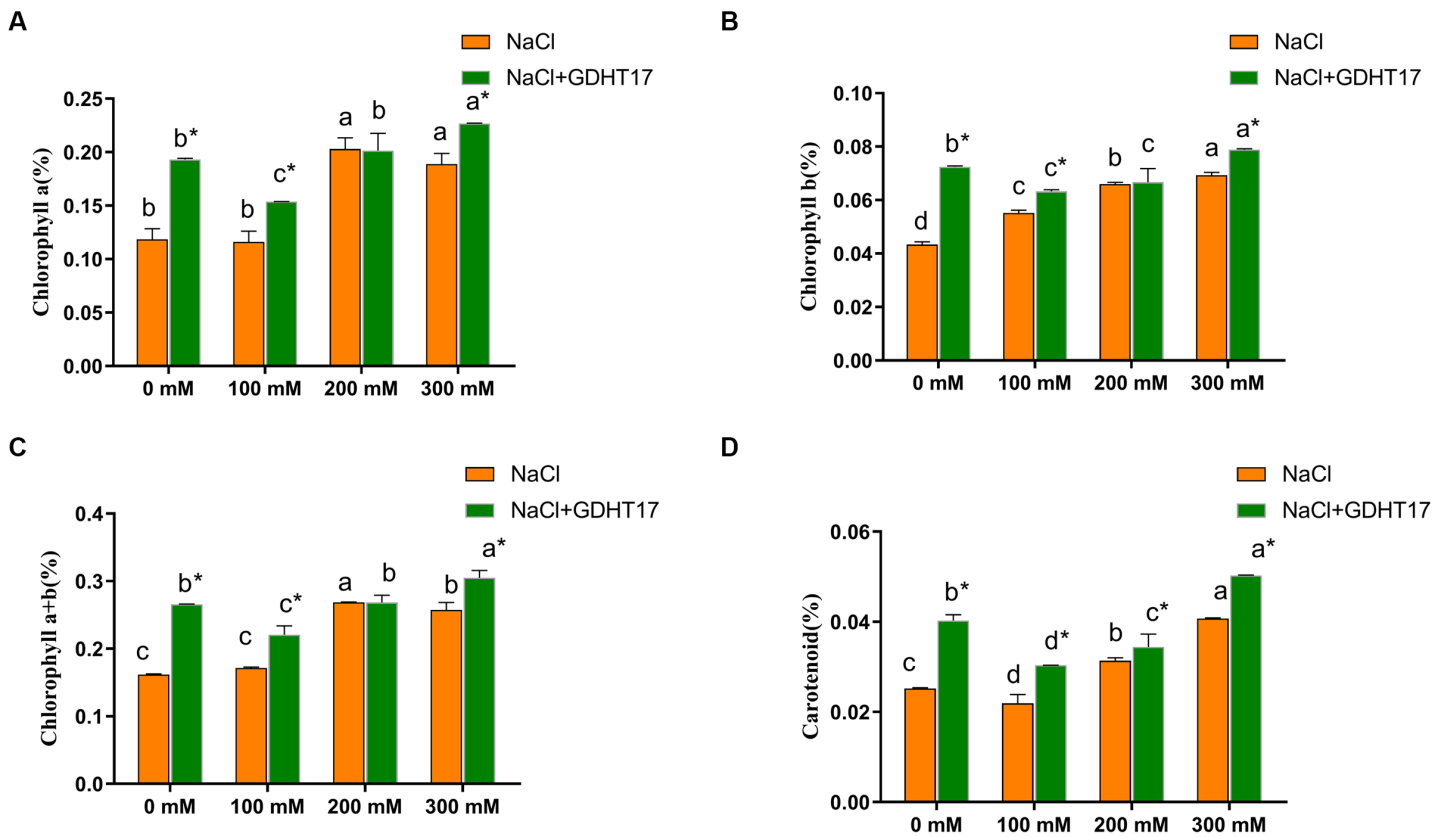
Inoculation of *G. dipsosauriuri* GDHT17 the chlorophyll content of NaCl-treated ryegrass seedlings. **(A)** Chlorophyll a content, **(B)** chlorophyll b content, **(C)** chlorophyll total content, **(D)** carotenoid content. Bar diagrams represent the mean of three replicates (*n* = 3). Error bars indicate the standard deviation (SD) of the three replicates. Different lowercase letters mean NaCl or NaCl+GDHT17; there were significant differences when different concentrations of NaCl treatments (*p* < 0.05). Bars diagrams marked with “*” indicate significant differences between NaCl and NaCl+GDHT17 treatment in the same NaCl concentration (*p* < 0.05).

Salt stress significantly increases the chlorophyll and carotenoid contents of ryegrass seedlings treated with 200 mM and 300 mM NaCl. Inoculating of GDHT17 significantly increased the contents of chlorophyll a, chlorophyll b, total chlorophyll, and carotenoids at the non-salt stress by 62.97, 66.96, 64.22, and 59.54%, respectively, compared to the non-GDHT17 treatment. Inoculating of GDHT17 significantly increased the carotenoid contents at the salt stress of 100 mM, 200 mM, and 300 mM NaCl and increased the chlorophyll contents at the salt stress of 100 mM and 300 mM NaCl (Figure 5).

### 3.5. Inoculation of GDHT17 affected the contents of malondialdehyde and proline

**Figure 6:**
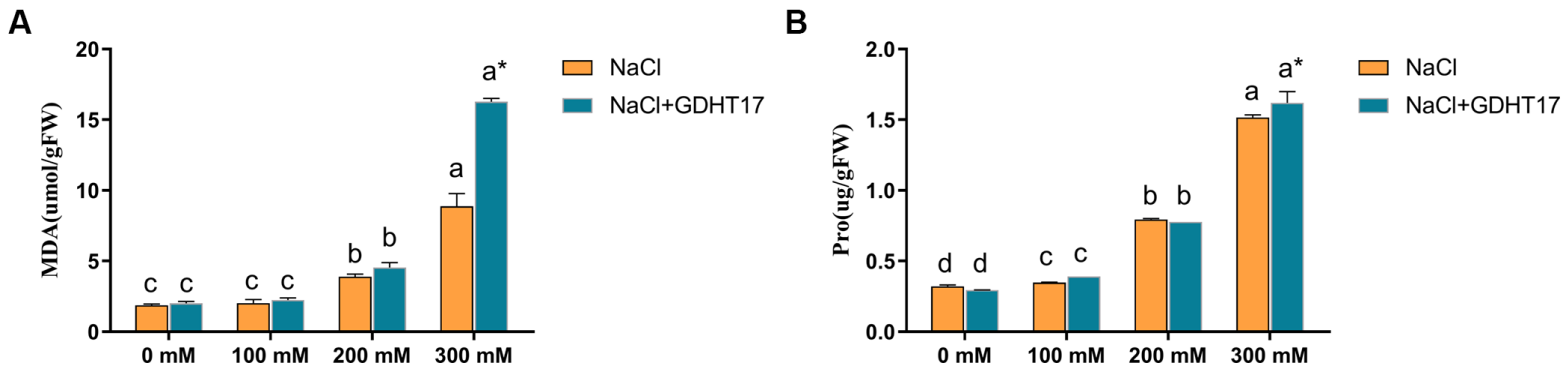
Inoculation of *G. dipsosauri* GDHT17 affected stressor metabolites of NaCl-treated ryegrass seedlings. **(A)** Malondialdehyde (MDA), **(B)** proline (pro). Bar diagrams represent the mean of three replicates (*n* = 3). Error bars indicate the standard deviation (SD) of the three replicates. Different lowercase letters mean NaCl or NaCl+GDHT17; there were significant differences when different concentrations of NaCl treatments (*p* < 0.05). Bars diagrams marked with “*” indicate significant differences between NaCl and NaCl+GDHT17 treatment in the same NaCl concentration (*p* < 0.05).

Salt stress significantly increased the contents of MDA and Pro in the ryegrass seedlings, especially in the seedlings treated with 200 mM and 300 mM NaCl, and also increased the contents of Pro in the seedlings treated with 100 mM (Figure 6). Inoculation with GDHT17 significantly increased the contents of MDA and Pro by 83.50 and 6.87% at the salt stress of 300 mM NaCl, compared to the non-GDHT17 treatment. However, the effect on the contents of MDA and Pro was not evident at the salt stress of 100 mM and 200 mM NaCl, even inoculating with GDHT17. Therefore, we can conclude that GDHT17 alleviates the NaCl-induced toxicity and improved proline and MDA content in ryegrass via the upregulation of antioxidant defense enzymatic activities (Figure 6).

### 3.6. Inoculation of GDHT17 affected ion absorption

**Figure 7:**
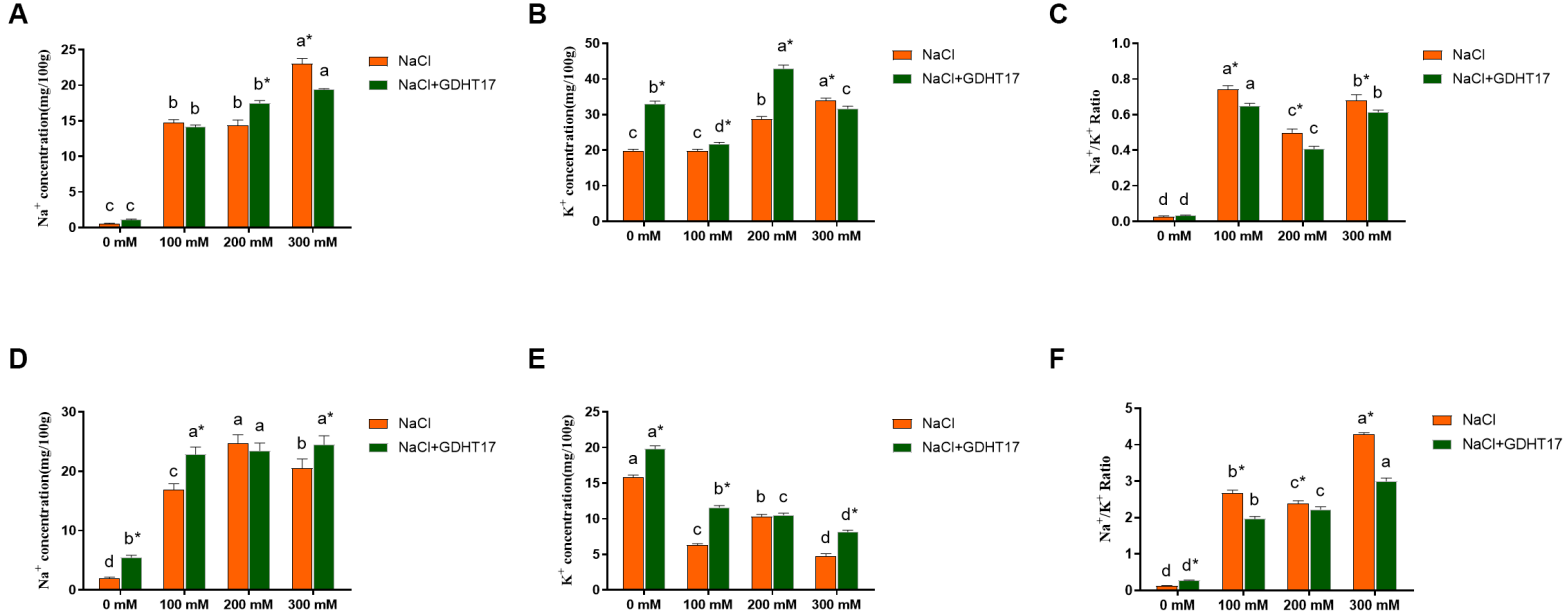
Inoculation of *G. dipsosauri* GDHT17 Affected Ion absorption in ryegrass seedlings. **(A)** Na^+^ content in leaves, **(B)** K^+^ content in leaves, **(C)** Na^+^/ K^+^ ratio in leaves, **(D)** Na^+^ content in roots, **(E)** K^+^ content in roots, **(F)** Na^+^/ K^+^ ratio in roots. Bar diagrams represent the mean of three replicates (*n* = 3). Error bars indicate the standard deviation (SD) of the three replicates. Different lowercase letters mean NaCl or NaCl+GDHT17; there were significant differences when treated with different concentrations of NaCl (*p* < 0.05). Bars diagrams marked with “*” indicate significant differences between NaCl and NaCl+GDHT17 treatment in the same NaCl concentration (*p* < 0.05).

Compared to the non-salt stress, salt stress caused a significant accumulation of Na^+^ and K^+^ contents and a significant increase of Na^+^/K^+^ ratio in ryegrass seedling leaves (Figures 7A–C); and caused a significant accumulation of Na^+^ content, a significant reduction of K^+^ content, and a significant increase of Na^+^/K^+^ ratio in seedling roots (Figures 7D–F). Compared to the non-GDHT17 inoculation, the Na^+^/K^+^ ratio significantly decreased by 12.45, 18.26, and 9.74% in the GDHT17-inoculated seedling leaves treated with 100 mM, 200 mM, and 300 mM NaCl, respectively. The Na^+^/K^+^ ratio significantly decreased by 26.52, 6.89, and 29.92% in the GDHT17-inoculated seedling roots treated with 100 mM, 200 mM, and 300 mM NaCl, respectively, compared to the non-GDHT17 treatment. Overall, GDHT17 decreases the Na^+^/K^+^ ratio under salt stress, whether in seedling leaves or roots, indicating the alleviating effect of GDHT17 on salt stress to ryegrass.

### 3.7. GDHT17 Modulated the antioxidant defense enzymes of NaCl-treated ryegrass

**Figure 8:**
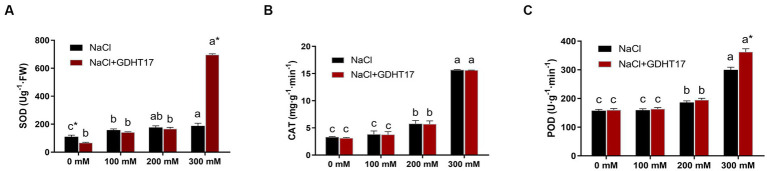
Inoculation of *G. dipsosauri* GDHT17 affected antioxidant defense enzymes of NaCl-treated ryegrass seedlings. **(A)** superoxide dismutase (SOD), **(B)** catalase (CAT), and **(C)** peroxidase (POD). Bar diagrams represent the mean of three replicates (*n* = 3). Error bars indicate the standard deviation (SD) of the three replicates. Different lowercase letters mean NaCl or NaCl+GDHT17; there were significant differences when treated with different concentrations of NaCl (*p* < 0.05). Bars diagrams marked with “*” indicate significant differences between NaCl and NaCl+GDHT17 treatment in the same NaCl concentration (*p* < 0.05).

Salt stress significantly increased the POD, CAT, and SOD activity of ryegrass seedlings under salt stress, especially those treated with 200 mM and 300 mM NaCl. The activities of the three antioxidant defense enzymes further increased in the GDHT17-inoculated seedlings. Compared with the non-GDHT17 treatment, the POD and SOD activity of the GDHT17-inoculated seedlings significantly increased by 25.83 and 250.79%, respectively, at a salt stress of 300 mM NaCl. In addition, the activity of POD, CAT, and SOD did not show a significant increasing effect at the NaCl salt stress of 100 mM and 200 mM concentrations. The activity of CAT did not significantly increase in the GDHT17-inoculated seedlings. It suggested that GDHT17 reduces salt damage to the ryegrass seedlings by eliminating ROS in ryegrass seedlings, especially under higher salt stress (Figure 8).

## 4. Discussion

As available natural bioresources, bacteria habited in the saline environment have been verified to have great potential application in stimulating the growth of salt-sensitive crops and promoting salt stress tolerance (Egamberdieva and Kucharova, 2009; Etesami and Beattie, 2017). In the study, a moderately halophilic bacterium *G. dipsosauri* GDHT17 was isolated from the soils collected from the Yellow River Delta in Shandong Province, China. The Yellow River Delta is a fluvial delta plain in the Yellow River estuary. It is one of the specific regions in China, where severe land salinization ecological conditions and environment are marked. Yellow River Delta is between land and sea and lacks fresh water, which results in an excessively high salt content that contributes to various degrees of salinization (Bian et al., 2021). The benefit of *G. dipsosauri* GDHT17 with alleviating salt-stress damage properties on ryegrass verified the challenges to obtaining such bacteria from those salinization habits. It also demonstrated the opportunities of applying these bacteria in salinization soils for soil amelioration.

Bacteria of *Gracilibacilluss* genus are moderately or heavily salinophilic (Vreeland et al., 2000; Fendrihan et al., 2006; Egamberdieva et al., 2019). *Gracilibacillus* formerly belonged to the genus *Bacillus* and was labeled as a salt stress-adapted, endospore-forming bacterium because of its specific physiological properties (Lawson et al., 1996; Vreeland et al., 2000). The genus now includes eight species with validly published names: *G. halotolerans*, *G. dipsosauri*, *G. orientalis*, *G. lacisalsi*, *G. saliphilus*, *G. boraciitolerans*, *G. halophilush*, and *G. quinghaiensi* (Carrasco et al., 2006; Ahmed et al., 2007; Chen et al., 2008; Jeon et al., 2008; Tang et al., 2009). Depending on the optimal salt requirement for growth, bacteria are classified as-slight (1.99–4.97% NaCl), moderate (4.97–19.89%), and extreme halophiles (19.89–29.84% NaCl) (Abaramak et al., 2020). These bacteria of the *Gracilibacilluss* genus are optimal salt growth at 5 to 20% NaCl concentration and are moderately or severely halophiles. *G. dipsosauri* GDHT17 is suitable for growth at 5% NaCl concentration, so it is considered a moderate halophilic bacterium. In the study, the potential of *G. dipsosauri* GDHT17 for promoting plant growth and for alleviating salt stress has been confirmed. It brings up prospective agents as bio-inoculants in the future. Moreover, we also carried out a preliminary identification of 159 isolates by 16srRNA analysis isolated from the saline soil samples collected from the Yellow River Delta (data not presented in this study). Most of which belonged to *Bacillus,* followed by *Pseudomonas*. These isolates collectively constitute the rich microbial resources in saline soils. Some of them probably are worth further study for exporting microorganisms for plant salt tolerance.

In this study, we suggested that *G. dipsosauriuri* GDHT17 positively affected the growth and biomass of NaCl-treated ryegrass. Inoculating GDHT17 can significantly promote ryegrass’s seedling height and stem diameter at different salt concentrations. The root length, diameter, and surface area of ryegrass seedlings also increased when the seedlings were inoculated with GDHT17. Research has suggested that salt stress mainly inhibits photosynthesis by reducing chlorophyll content and affecting photosystem II efficiency (Kalaji et al., 2011; Ma et al., 2012; Han et al., 2014). In this study, we found that the salt stress increased the chlorophyll content in ryegrass, while GDHT17 furtherly increased the chlorophyll content. The results suggest that the more effective increasing effect of GDHT17 on the chlorophyll content under salt stress, indicating the impact on the ryegrass copes with salt stress.

Proline is a stress molecule that protects organelles indicating protein damage and cell membranes from the adverse effects of increased concentrations of salts (Ashraf and Foolad, 2007). The increased production of Pro facilitates the maintenance of the balance of osmotic potential inside and outside the cell, thus increasing water intake and reducing plant damage due to salt stress (Islam et al., 2016; Shahid et al., 2022). Salinity-induced oxidative stress is often measured using lipid peroxidation, and malondialdehyde is a prevalent end product of lipid peroxidation (Villalobos-López et al., 2022). The results of the study also showed a significant increase in the contents of MDA and Pro in the ryegrass seedlings treated with salt stress of 200 mM and 300 mM NaCl, demonstrating salt damage caused by salt stress and a self-reducing damage mechanism of ryegrass seedlings. Inoculation with GDHT17 significantly increased the contents of MDA and Pro by 83.50 and 6.87% at the salt stress of 300 mM NaCl, indicating *G. dipsosauri* GDHT17 possesses an apparent function of alleviating the NaCl-induced toxicity on ryegrass by improving Pro and MDA content in ryegrass.

Plants exposed to salt stress tend to take up less Na^+^ and more K^+^, re-establishing ionic homeostasis (Zhu, 2001; Hu et al., 2012). Maintaining an appropriate Na^+^/K^+^ balance is considered an essential mechanism for plants to cope with salt stress (Mahajan and Tuteja, 2005; Evelin et al., 2009). In this study, salt stress caused a significant increase of Na^+^/K^+^ ratio in ryegrass seedling leaves and roots. Conversely, the Na^+^/K^+^ ratio significantly decreased in the ryegrass seedling leaves and roots inoculated with GDHT17. Some studies also demonstrated that the beneficial bacteria could reduce the Na^+^ uptake of plants, which helps to maintain a low Na^+^ /K^+^ ratio in the plants (Ashraf et al., 2004; Zhang et al., 2008; Dodd and Perez-Alfocea, 2012; Ali et al., 2019). Therefore, *G. dipsosauri* GDHT17 contributes to the alleviating effect of salt stress on ryegrass by maintaining an appropriate Na^+^/K^+^ balance.

Salt stress is usually associated with oxidative damage of reactive oxygen production. The accumulation of ROS in plant tissues leads to cell membrane damage as well as the oxidation of biomolecules (Li et al., 2019; Hasanuzzaman et al., 2020). Salt-induced ROS production in plants is counteracted by many enzyme scavengers, such as POD, CAT, SOD, or other antioxidants (Waller et al., 2005; Gururani et al., 2013; Shahid et al., 2022). As the concentration of NaCl in plants increases, the activity of antioxidant enzymes increases (Numan et al., 2018; Mubeen et al., 2022). Inoculating GDHT17 resulted in a more evident effect of promoting the activities of the antioxidant defense enzymes in ryegrass seedlings, and the SOD activity especially showed a dramatic increase of 250.79% under the salt stress of 300 mM NaCl. Our research suggests that *G. dipsosauri* GDHT17 plays a critical protective role in modulating the antioxidant defense enzymes of NaCl-treated ryeegrass, which counteracts oxidative damage and contributes to the salt tolerance of ryegrass.

## 5. Conclusion

In the study, we obtained a moderately halophilic bacteria *G. dipsosauri* GDHT17, from saline habitats in the Yellow River Delta, where severe land salinization is marked. We explored and verified the properties of the GDHT17 to alleviate the salt stress on perennial ryegrass. The results demonstrated that under salt stress conditions, GDHT17 benefits ryegrass growth, increases ryegrass biomass and water content, and increases chlorophyll and carotenoid content. *G. dipsosauri* GDHT17 possesses an apparent function of alleviating the NaCl-induced damage on ryegrass by improving Pro and MDA content in ryegrass, by maintaining an appropriate Na^+^/K^+^ balance and by modulating the antioxidant defense enzymes of NaCl-treated ryegrass to counteract oxidative damage, so that contributes to the salt tolerance of ryegrass. Therefore, we highlight the great potential of *G. dipsosauri* GDHT17 in protecting plants from salt stress.

## Data availability statement

The original contributions presented in the study are included in the article/Supplementary material, further inquiries can be directed to the corresponding author.

## Author contributions

XL, JZ, and ZL contributed to conception and design of the study. XL and ZG organized the database. WW performed the statistical analysis. XL wrote the first draft of the manuscript. ZL wrote sections of the manuscript. All authors contributed to the article and approved the submitted version.

## Funding

This research was supported by the National Natural Science Foundation of China (Nos. 31770685 and 31270687) and the Shandong Provincial Agricultural Science and Technology Fund Project (No. 2019LY003).

## Conflict of interest

The authors declare that the research was conducted in the absence of any commercial or financial relationships that could be construed as a potential conflict of interest.

## Publisher’s note

All claims expressed in this article are solely those of the authors and do not necessarily represent those of their affiliated organizations, or those of the publisher, the editors and the reviewers. Any product that may be evaluated in this article, or claim that may be made by its manufacturer, is not guaranteed or endorsed by the publisher.
