# Supplementary figures and images for: The halophilic bacteria Gracilibacillus dipsosauri GDHT17 alleviates salt stress on perennial ryegrass seedlings
Source: Front Microbiol. 2023 Jul 26;14:1213884. doi: 10.3389/fmicb.2023.1213884 (PMC10411512; doi:10.3389/fmicb.2023.1213884)

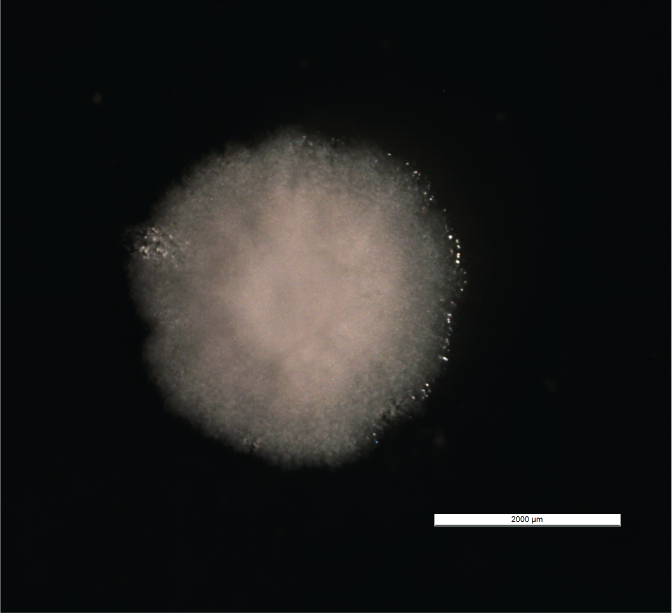

Supplement: Supplementary file 2 [file Data_Sheet_2.ZIP › Figure/Figure 1-A.tif]

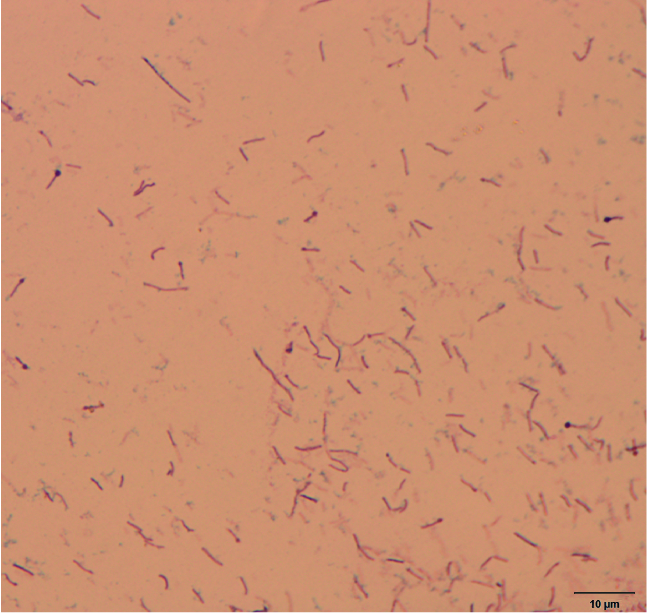

Supplement: Supplementary file 2 [file Data_Sheet_2.ZIP › Figure/Figure 1-B.tif]

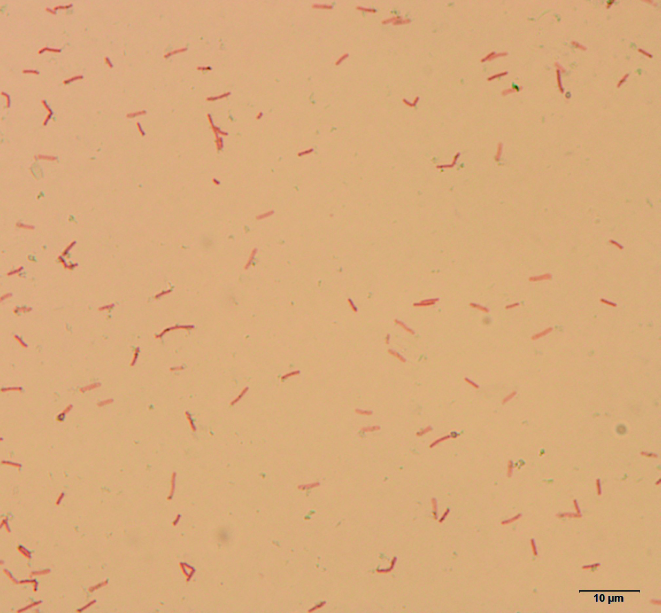

Supplement: Supplementary file 2 [file Data_Sheet_2.ZIP › Figure/Figure 1-C.tif]

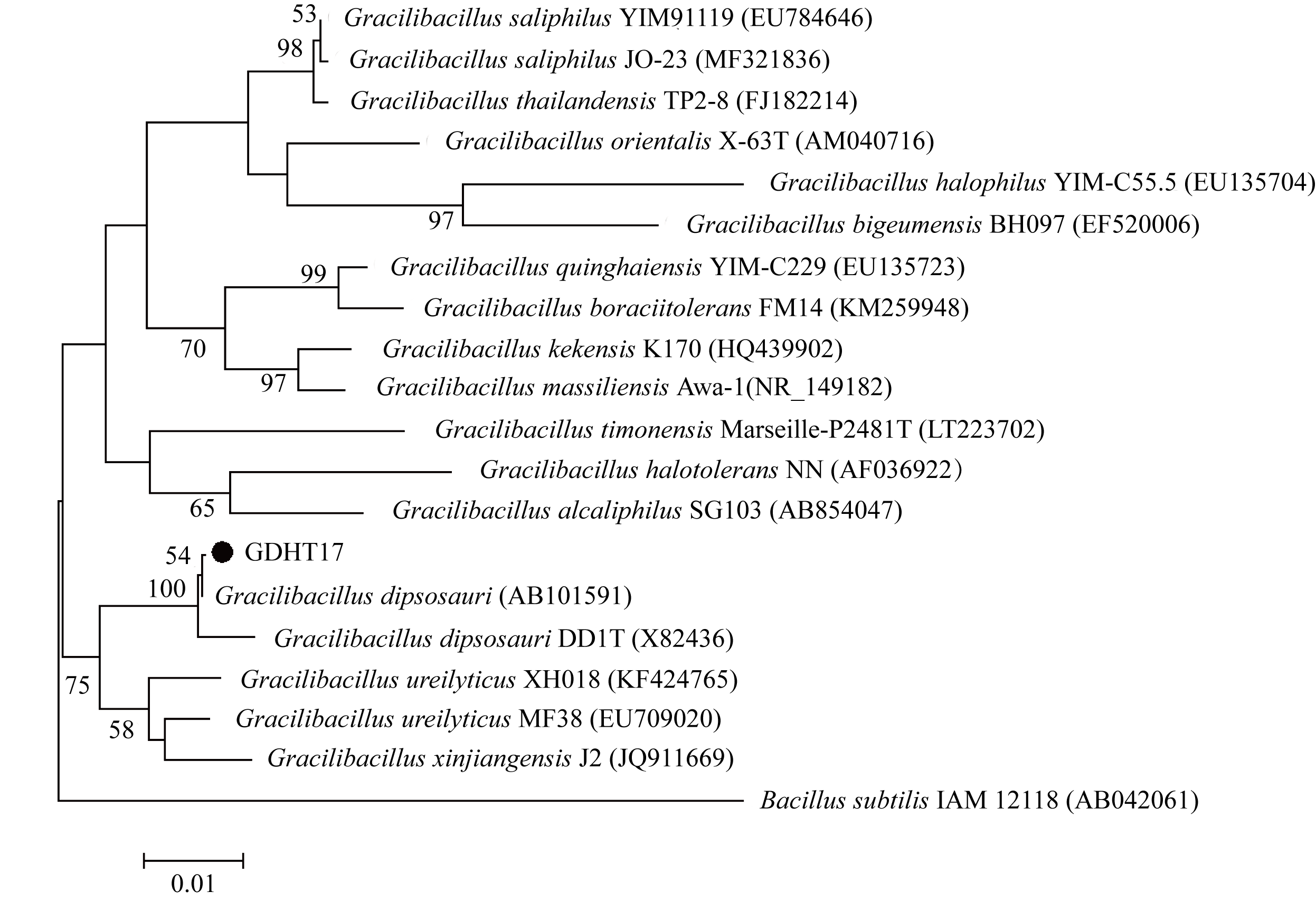

Supplement: Supplementary file 2 [file Data_Sheet_2.ZIP › Figure/Figure 2.tif]

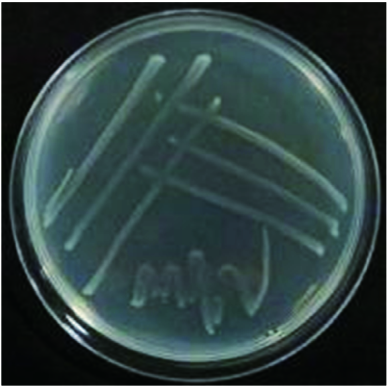

Supplement: Supplementary file 2 [file Data_Sheet_2.ZIP › Figure/Figure 3-A.tif]

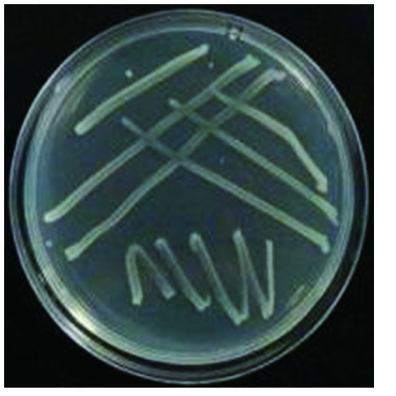

Supplement: Supplementary file 2 [file Data_Sheet_2.ZIP › Figure/Figure 3-B.tif]

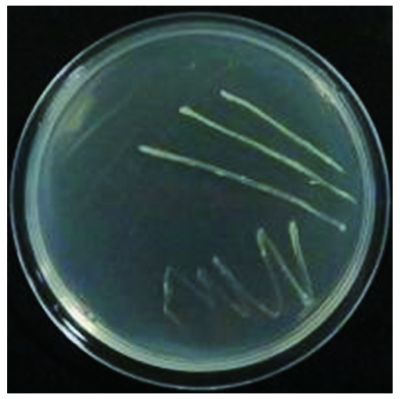

Supplement: Supplementary file 2 [file Data_Sheet_2.ZIP › Figure/Figure 3-C.tif]

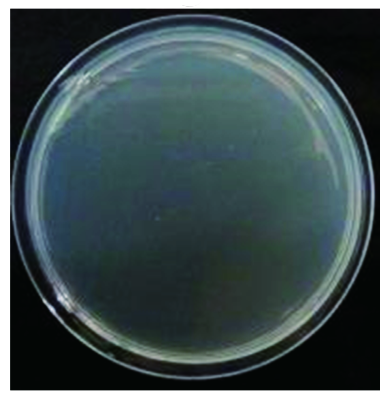

Supplement: Supplementary file 2 [file Data_Sheet_2.ZIP › Figure/Figure 3-D.tif]

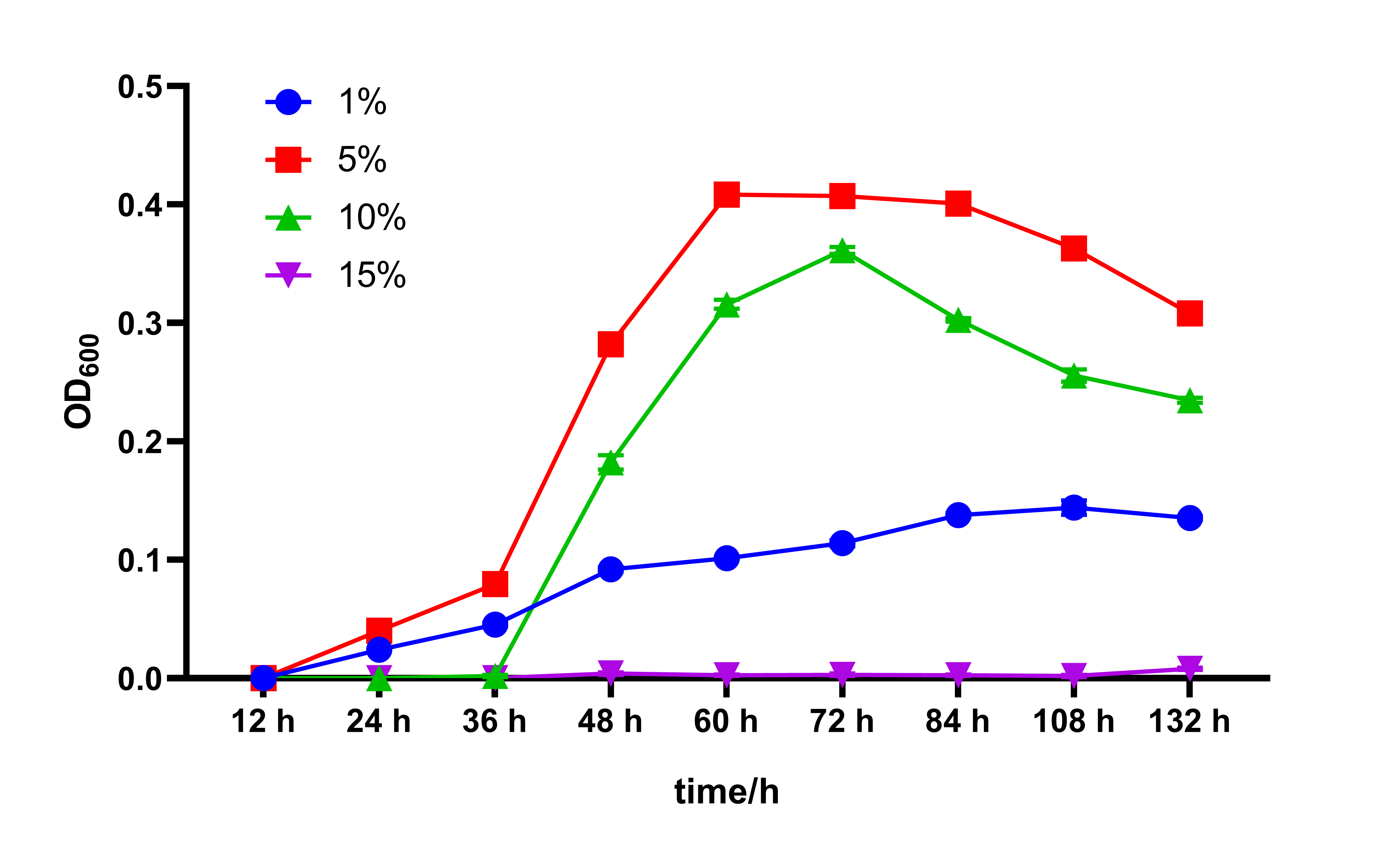

Supplement: Supplementary file 2 [file Data_Sheet_2.ZIP › Figure/Figure 3-E.tif]

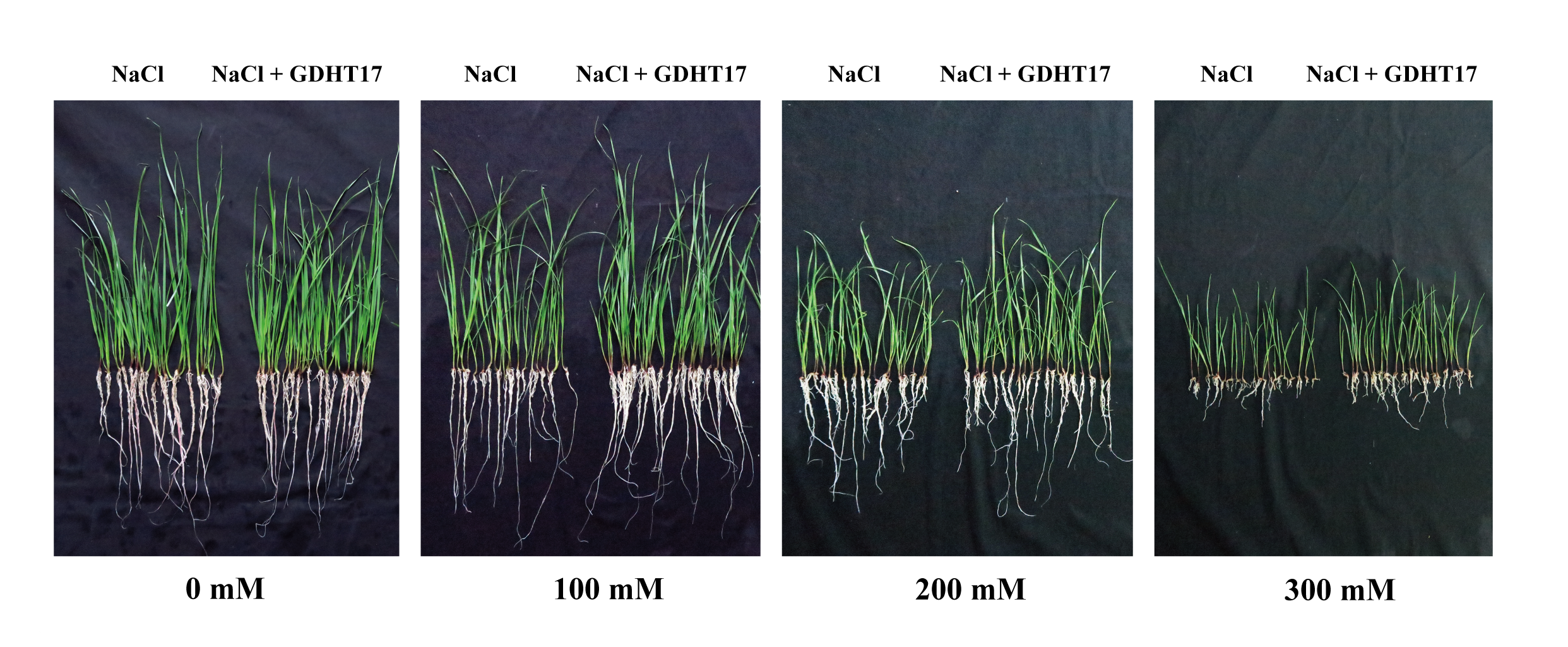

Supplement: Supplementary file 2 [file Data_Sheet_2.ZIP › Figure/Figure 4-A.tif]

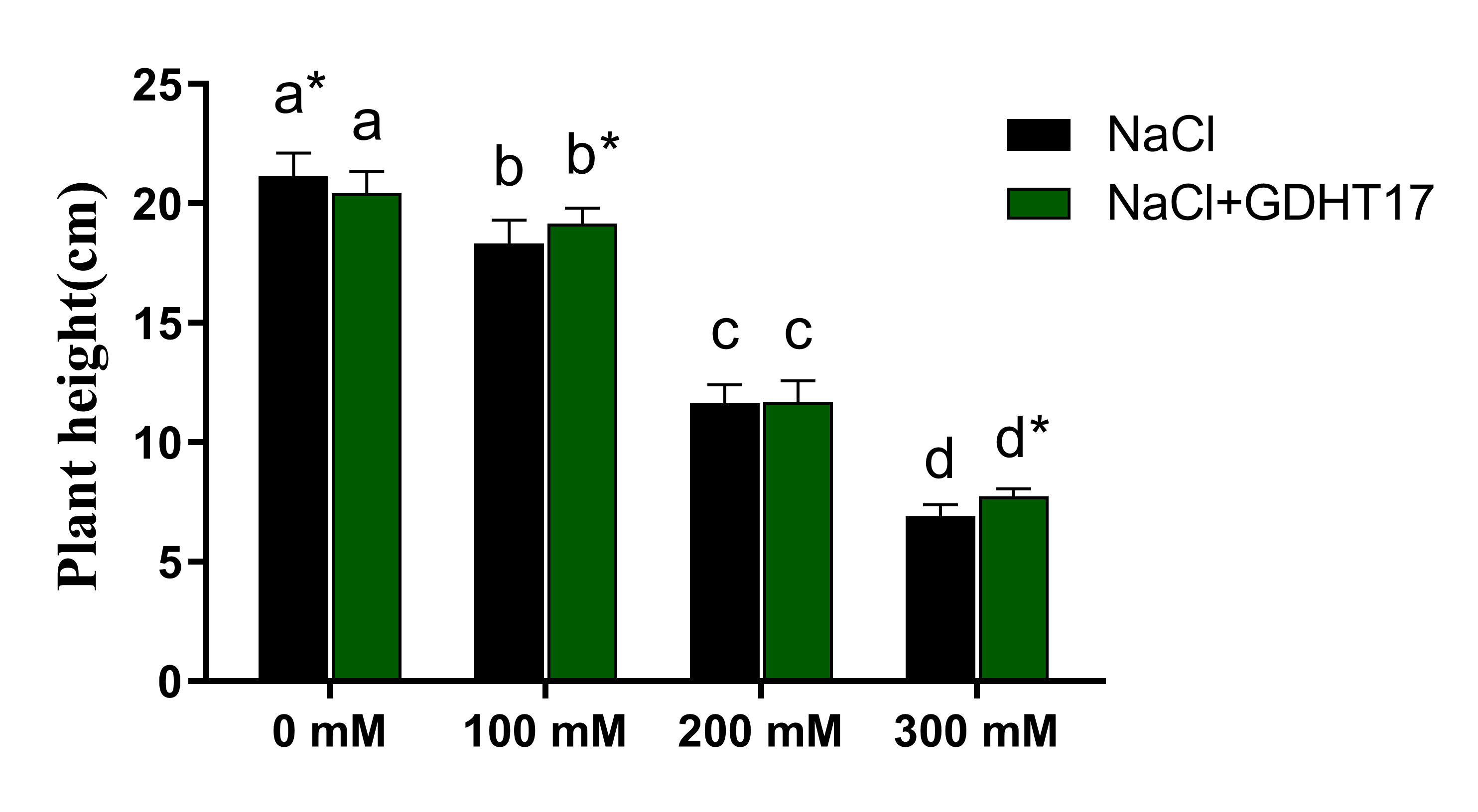

Supplement: Supplementary file 2 [file Data_Sheet_2.ZIP › Figure/Figure 4-B.tif]

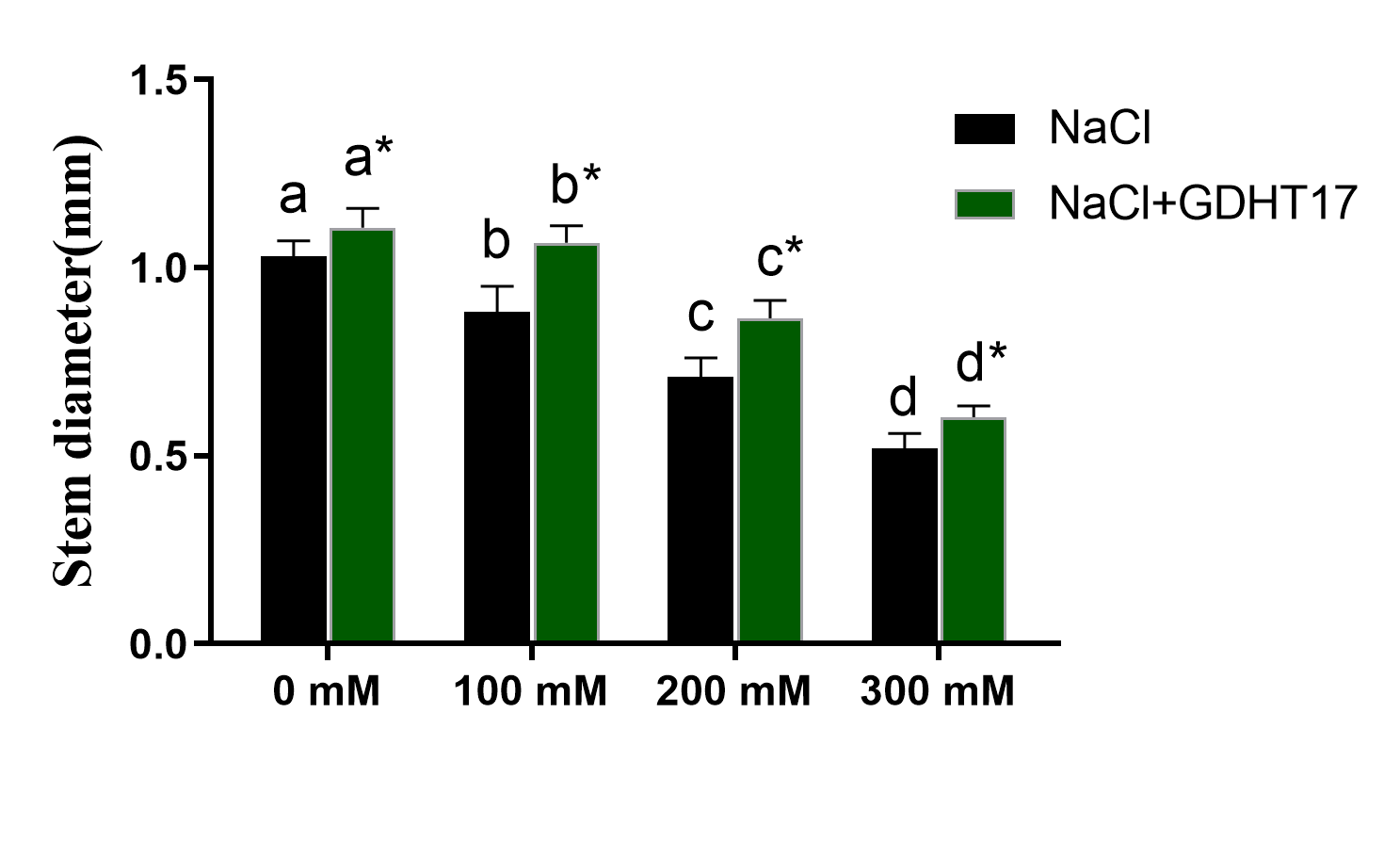

Supplement: Supplementary file 2 [file Data_Sheet_2.ZIP › Figure/Figure 4-C.tif]

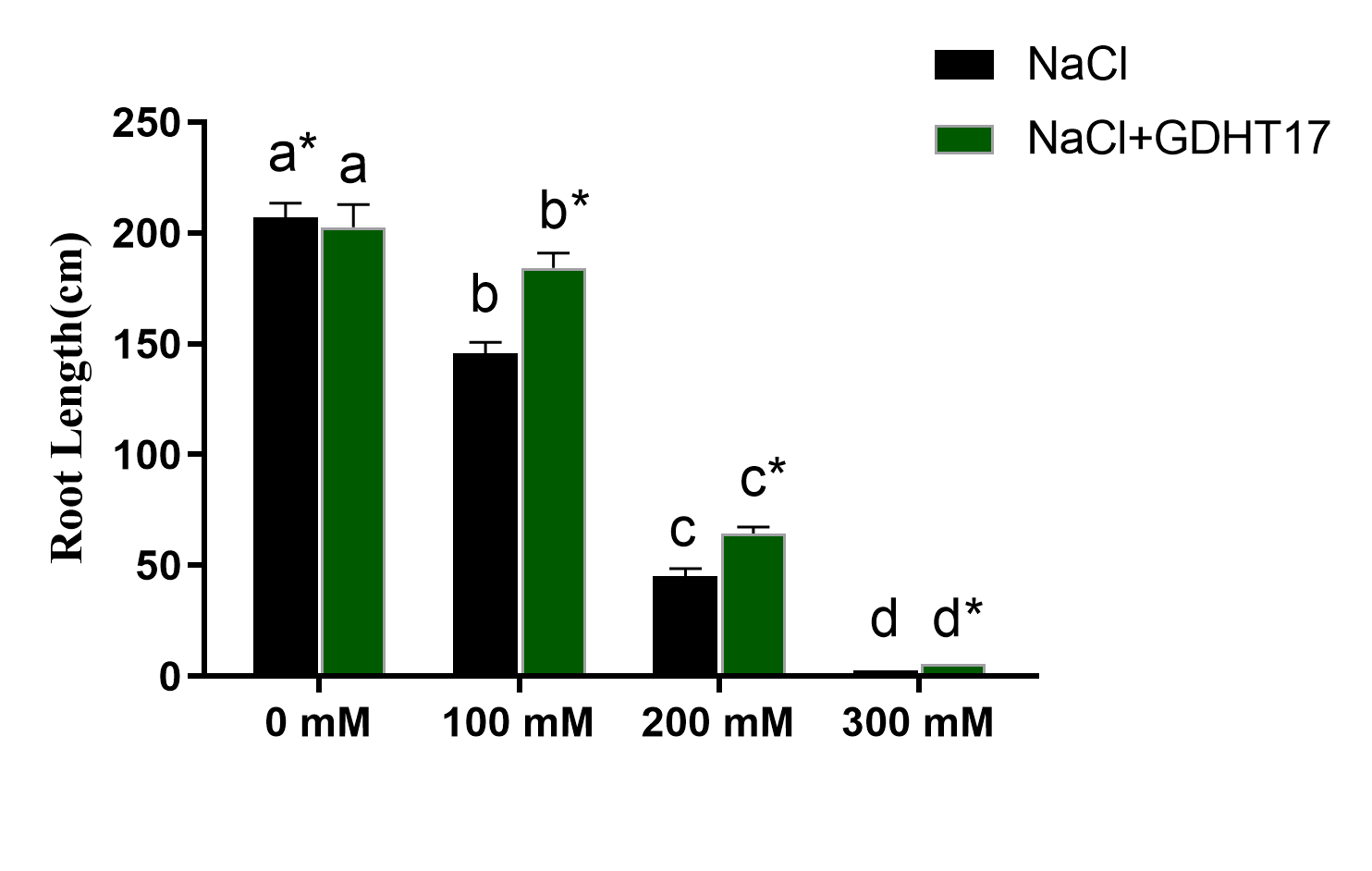

Supplement: Supplementary file 2 [file Data_Sheet_2.ZIP › Figure/Figure 4-D.tif]

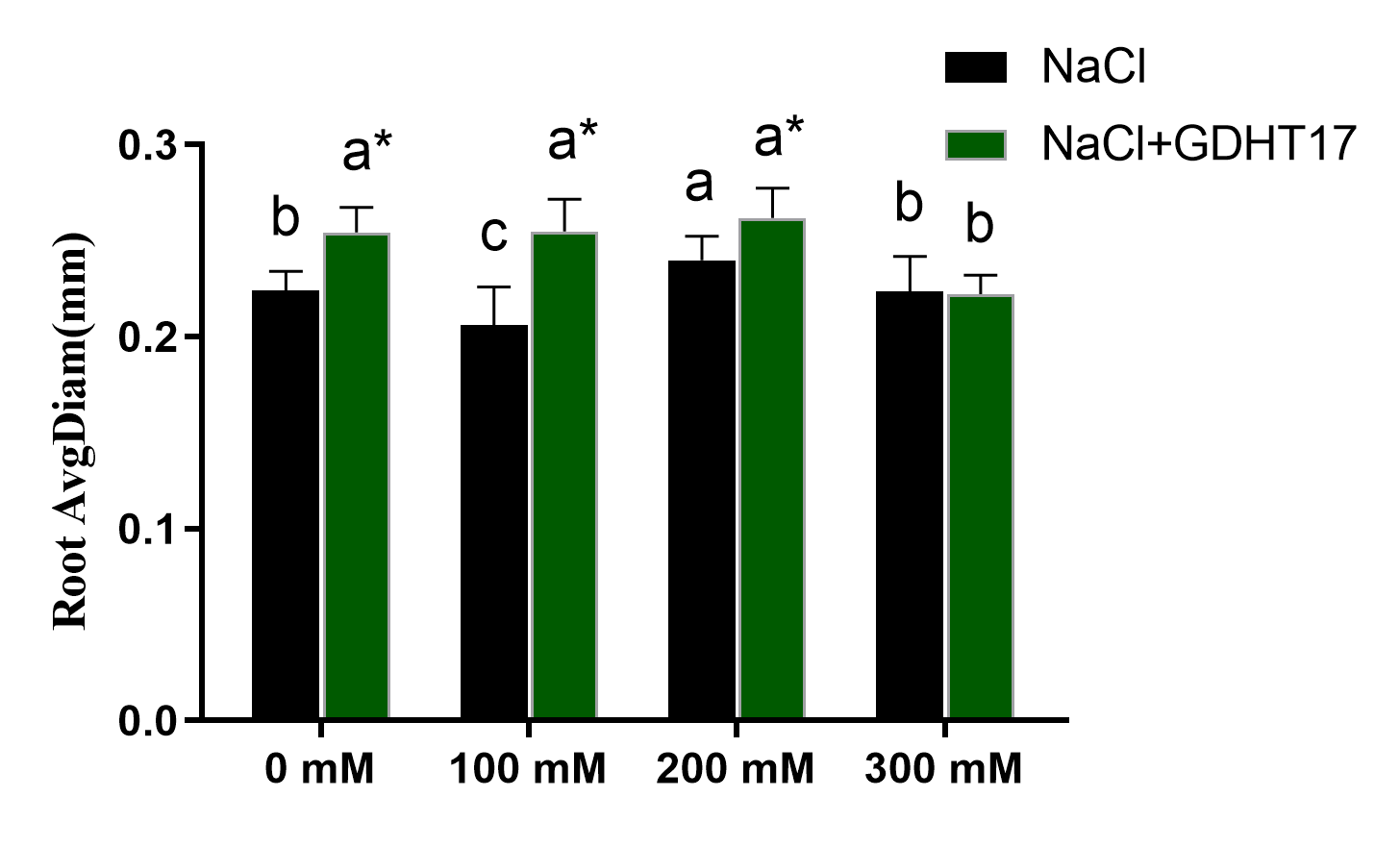

Supplement: Supplementary file 2 [file Data_Sheet_2.ZIP › Figure/Figure 4-E.tif]

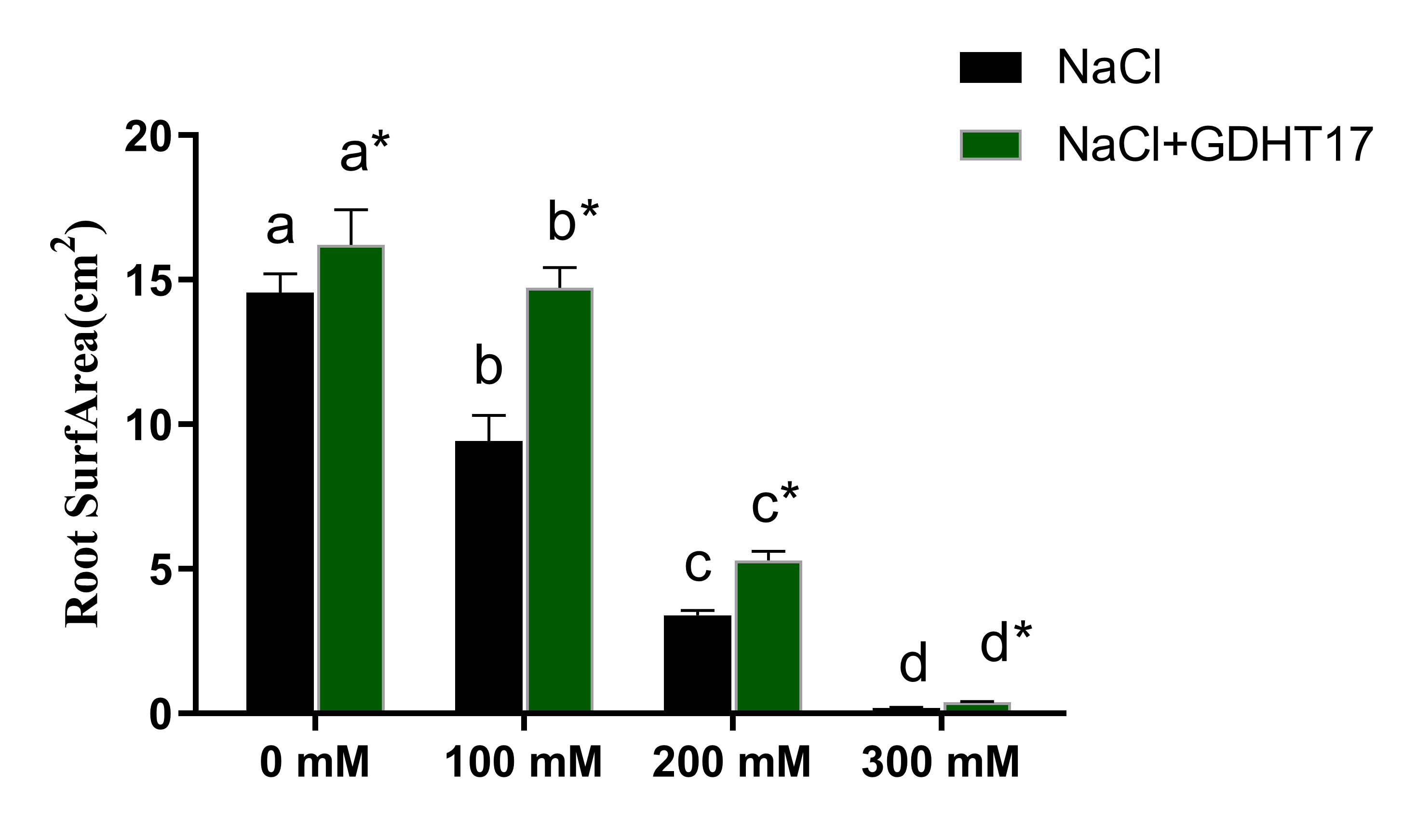

Supplement: Supplementary file 2 [file Data_Sheet_2.ZIP › Figure/Figure 4-F.tif]

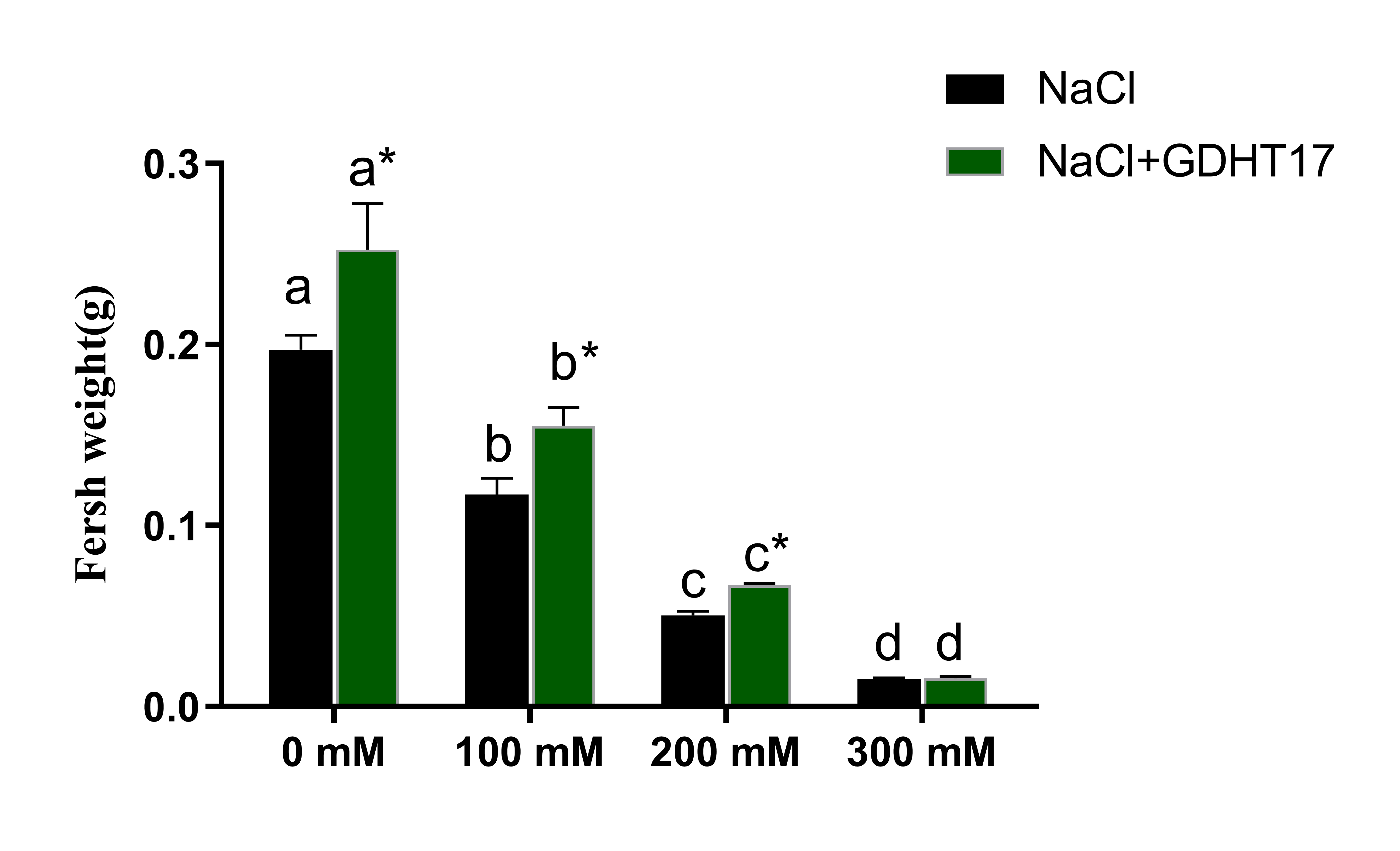

Supplement: Supplementary file 2 [file Data_Sheet_2.ZIP › Figure/Figure 4-G.tif]

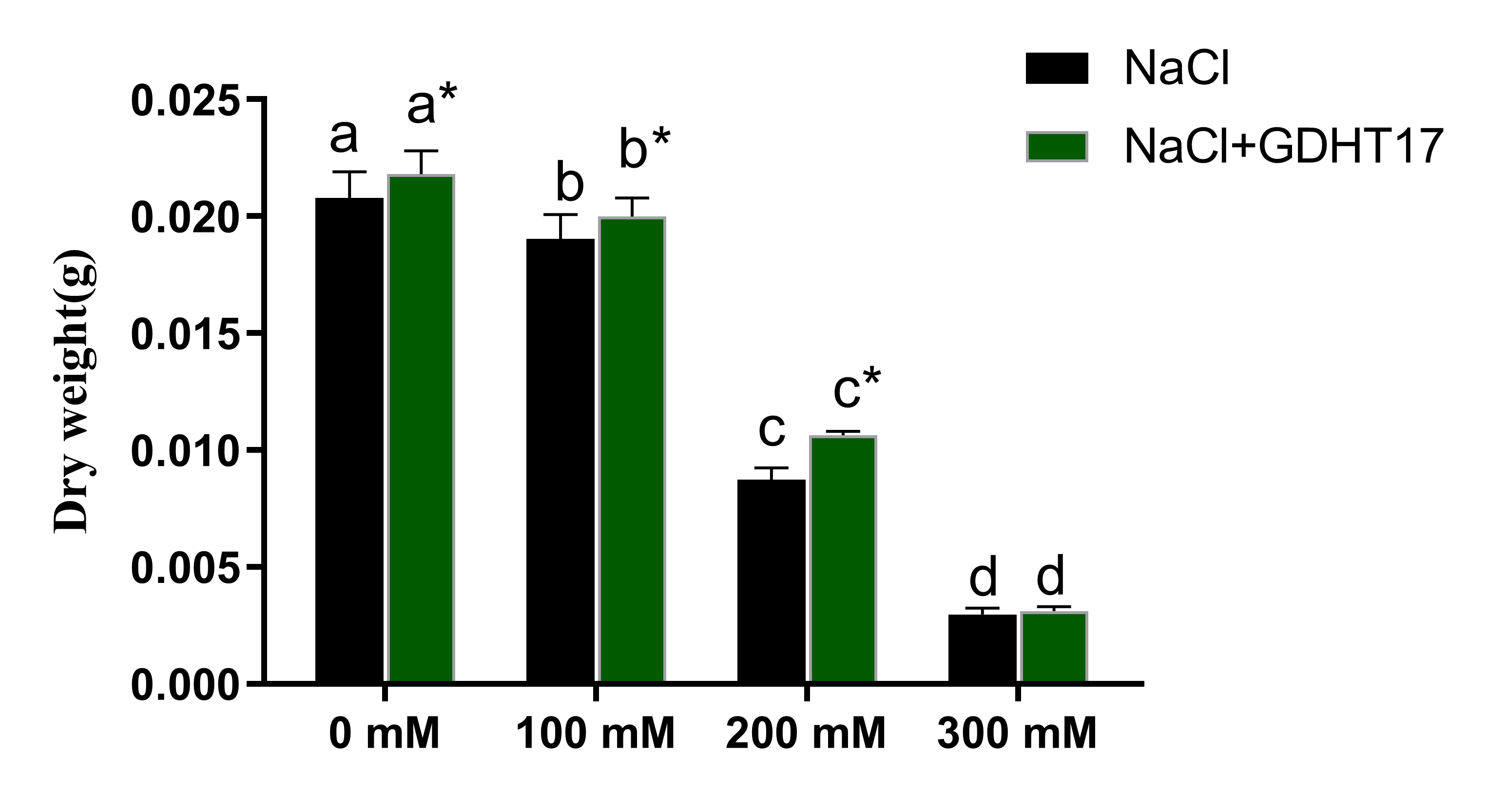

Supplement: Supplementary file 2 [file Data_Sheet_2.ZIP › Figure/Figure 4-H.tif]

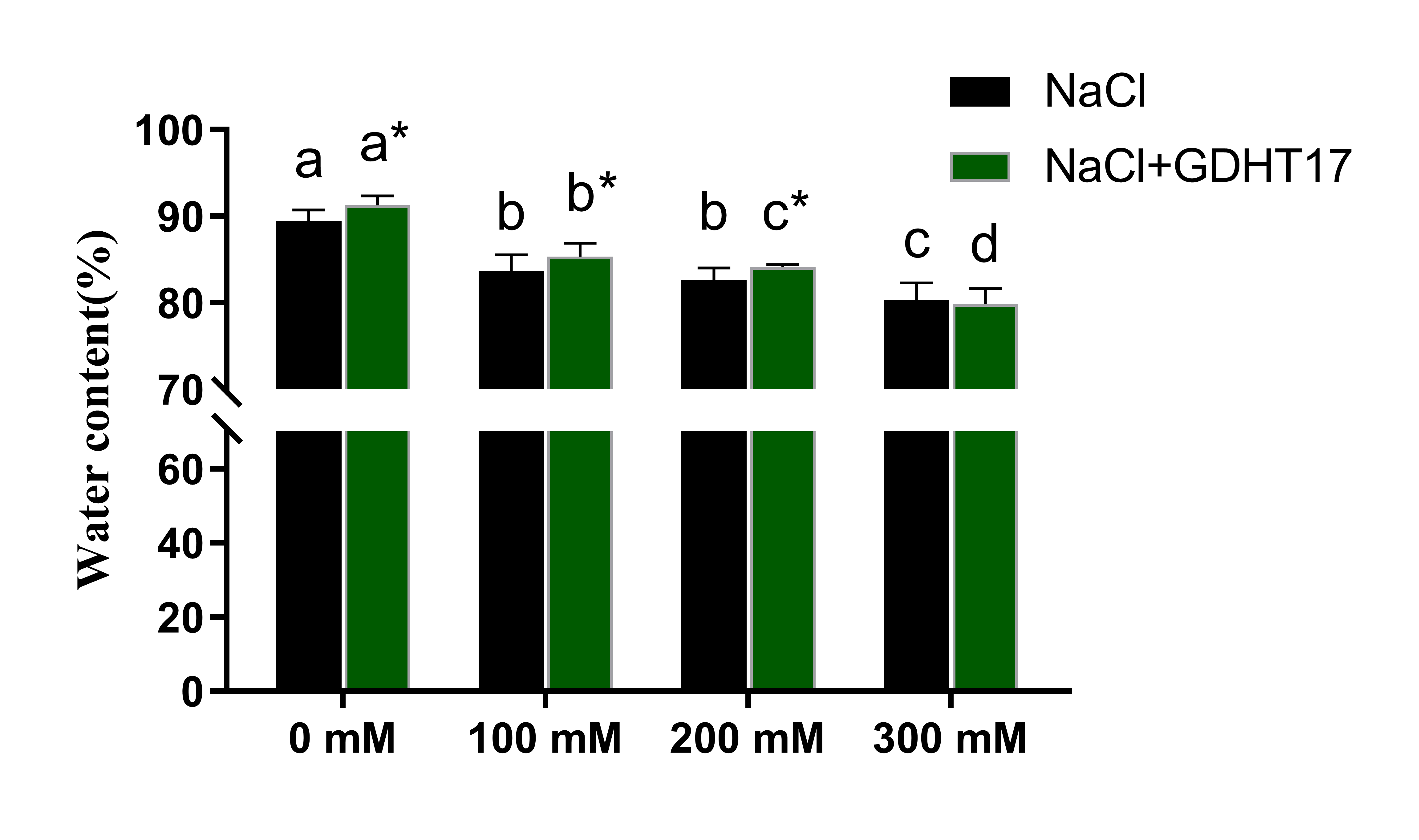

Supplement: Supplementary file 2 [file Data_Sheet_2.ZIP › Figure/Figure 4-I.tif]

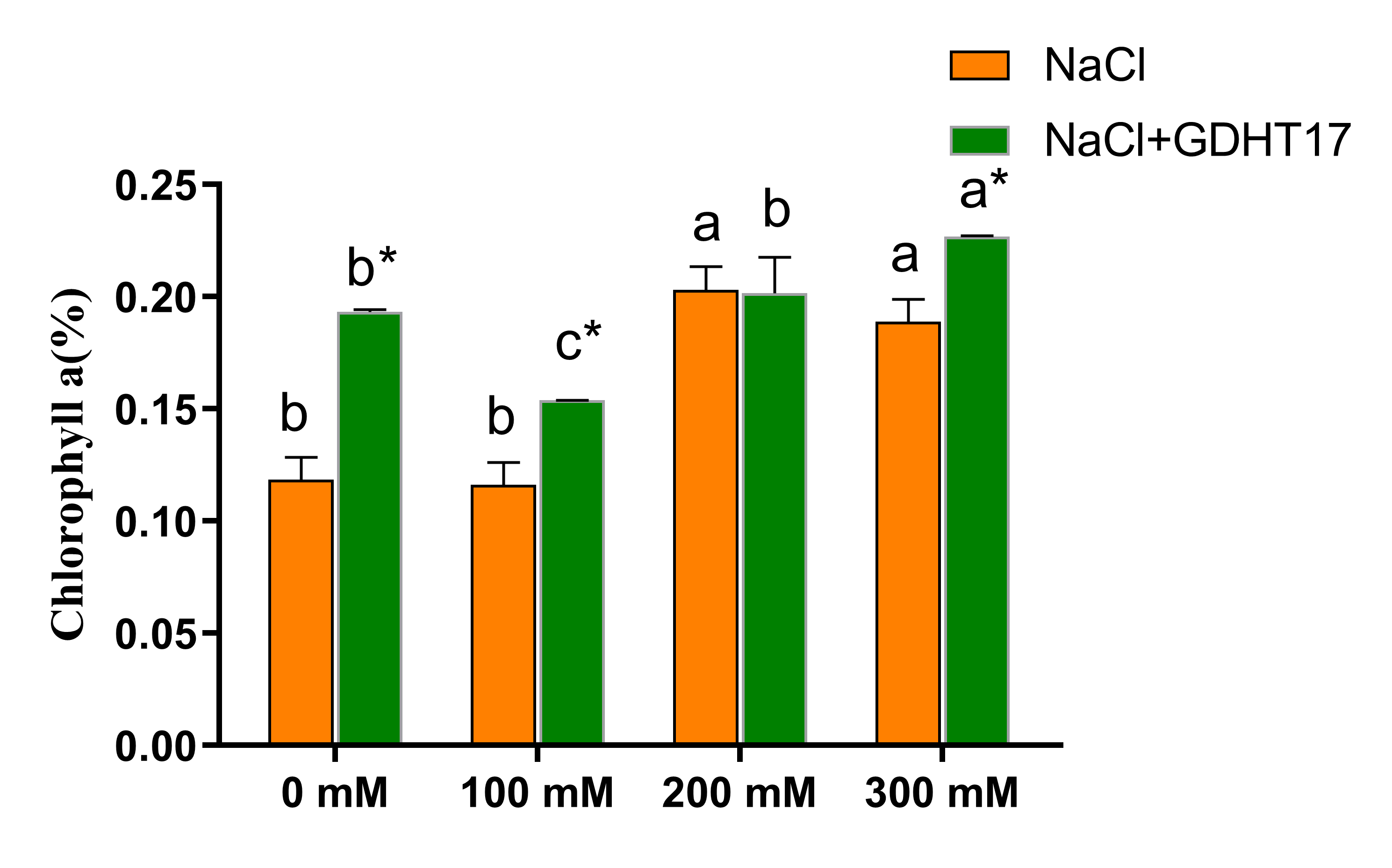

Supplement: Supplementary file 2 [file Data_Sheet_2.ZIP › Figure/Figure 5-A.tif]

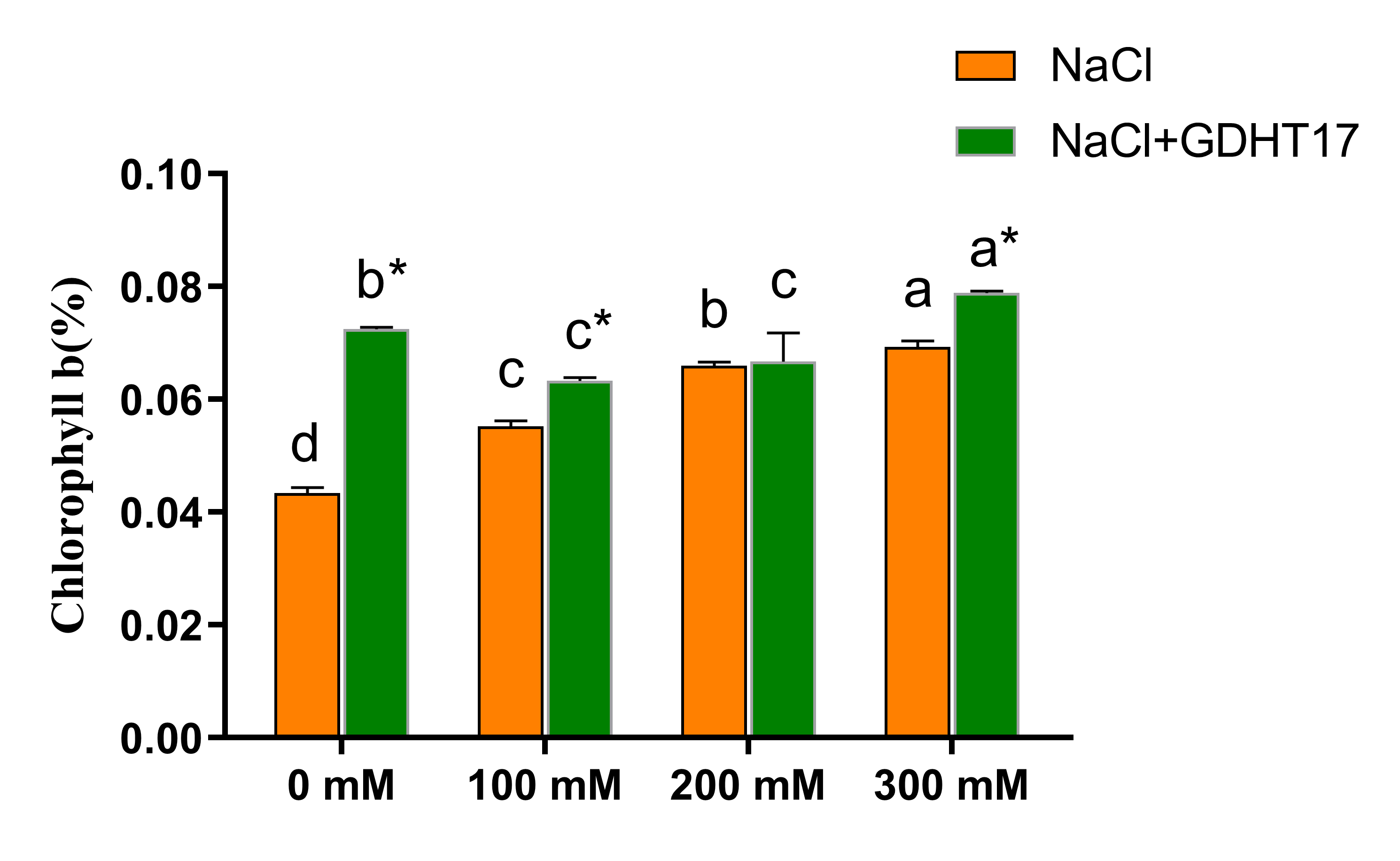

Supplement: Supplementary file 2 [file Data_Sheet_2.ZIP › Figure/Figure 5-B.tif]

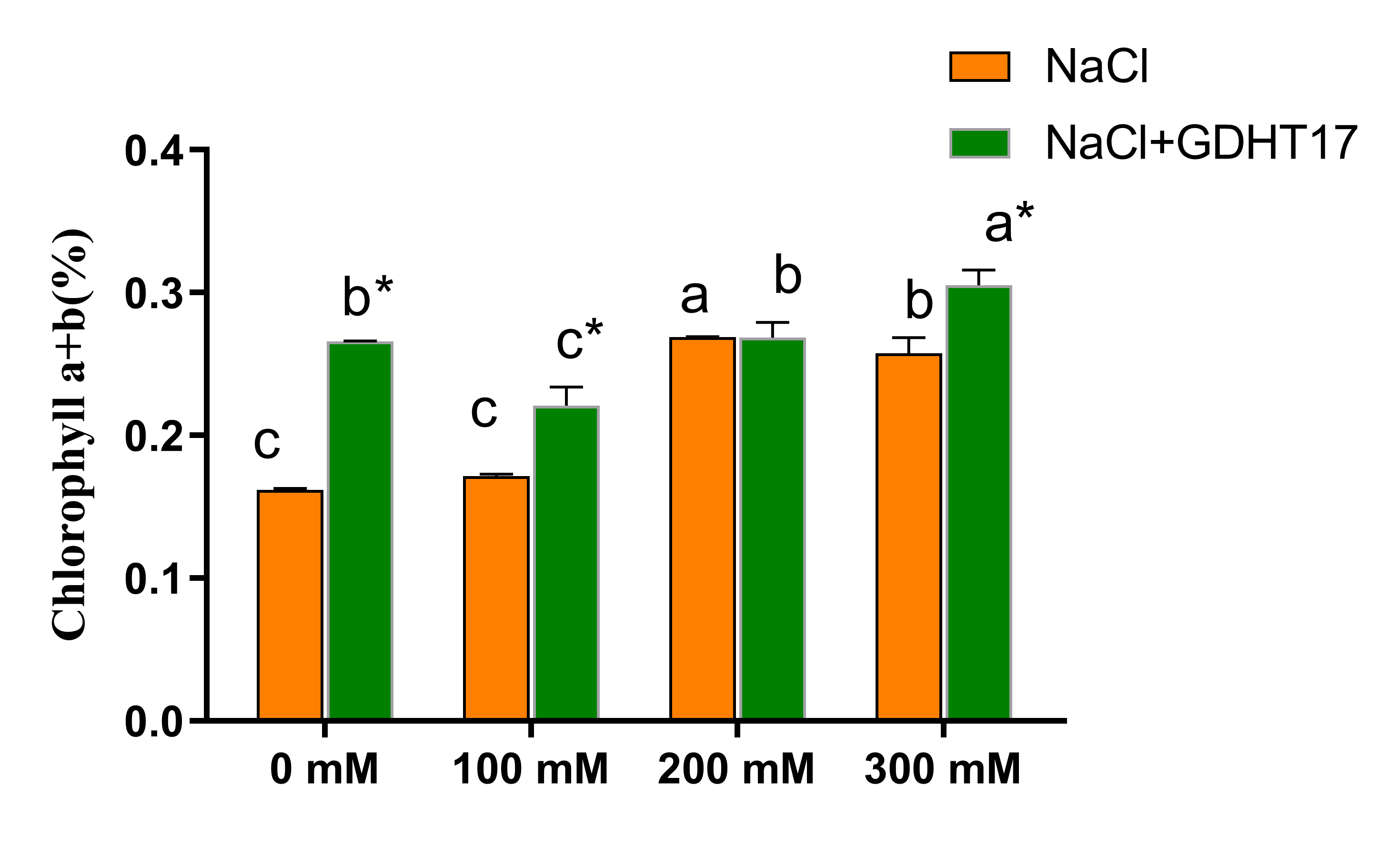

Supplement: Supplementary file 2 [file Data_Sheet_2.ZIP › Figure/Figure 5-C.tif]

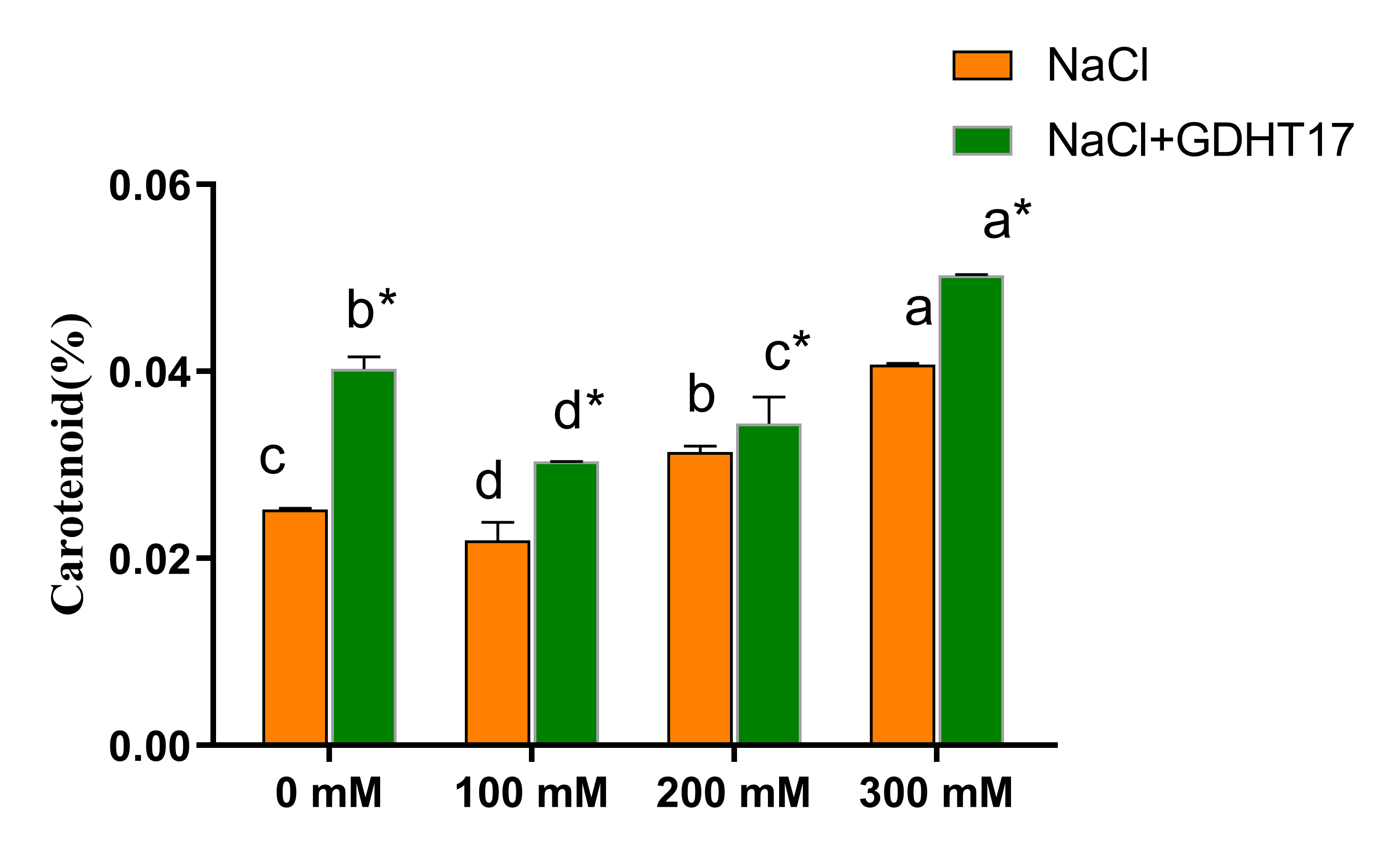

Supplement: Supplementary file 2 [file Data_Sheet_2.ZIP › Figure/Figure 5-D.tif]

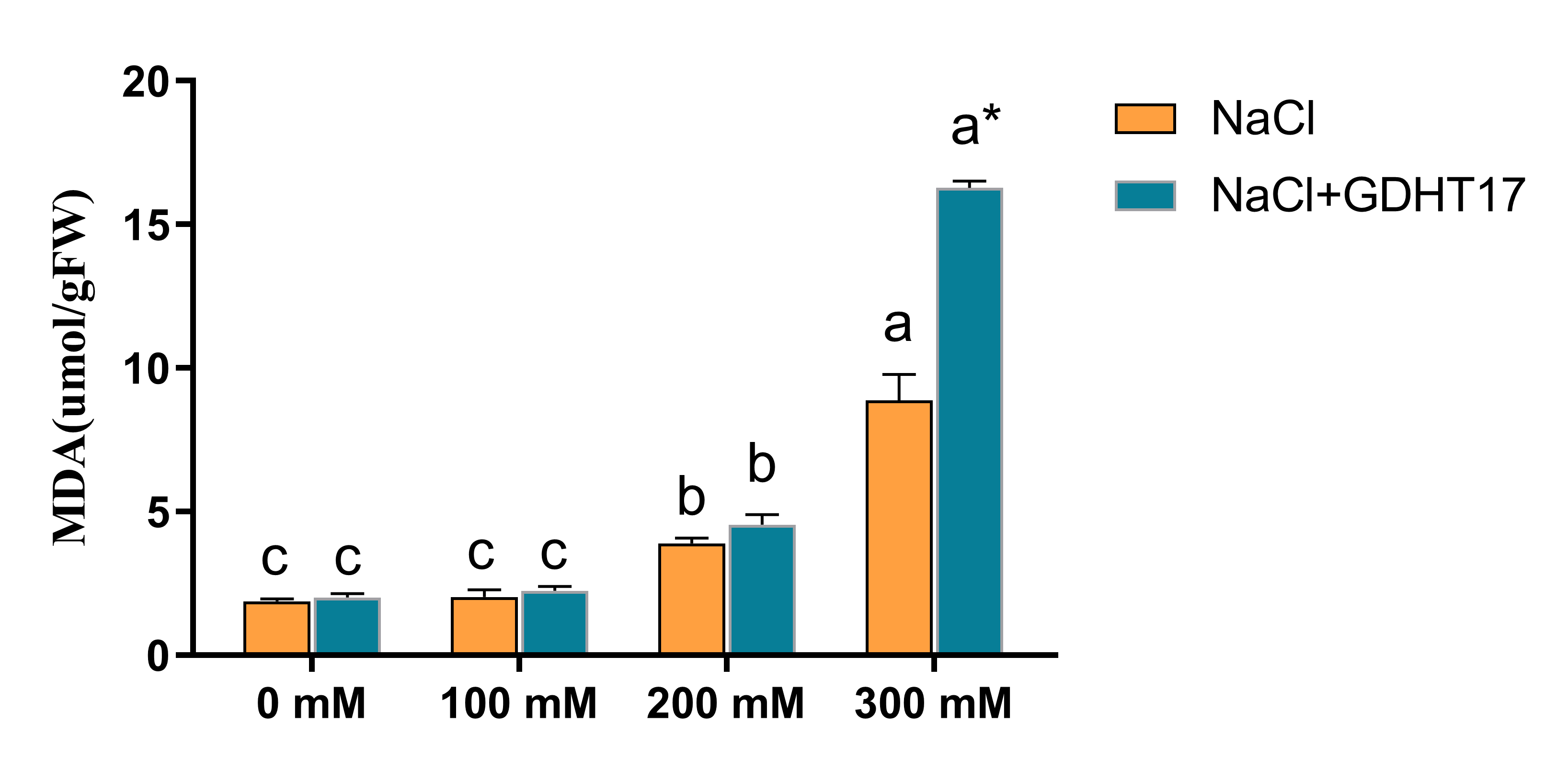

Supplement: Supplementary file 2 [file Data_Sheet_2.ZIP › Figure/Figure 6-A.tif]

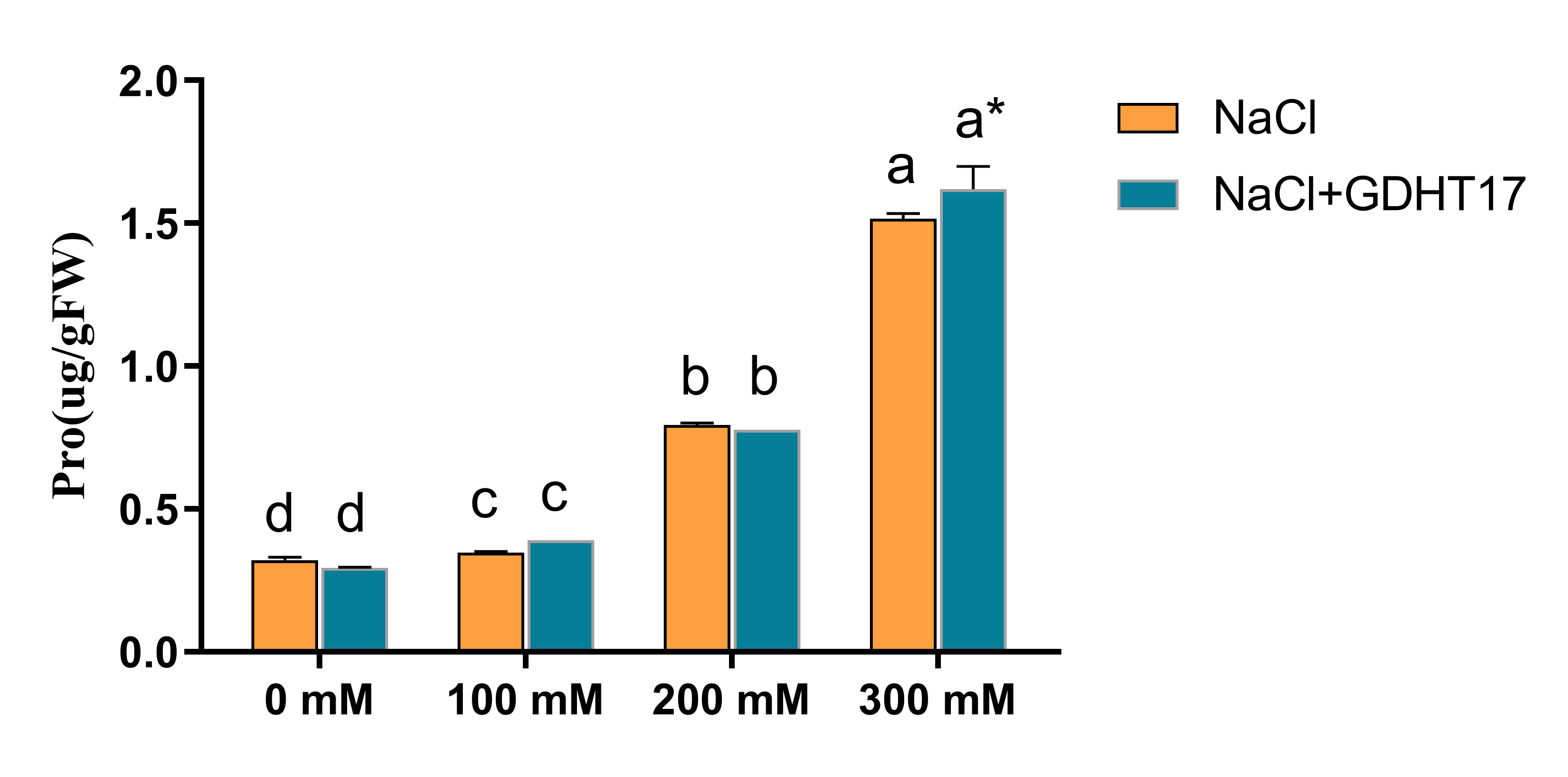

Supplement: Supplementary file 2 [file Data_Sheet_2.ZIP › Figure/Figure 6-B.tif]

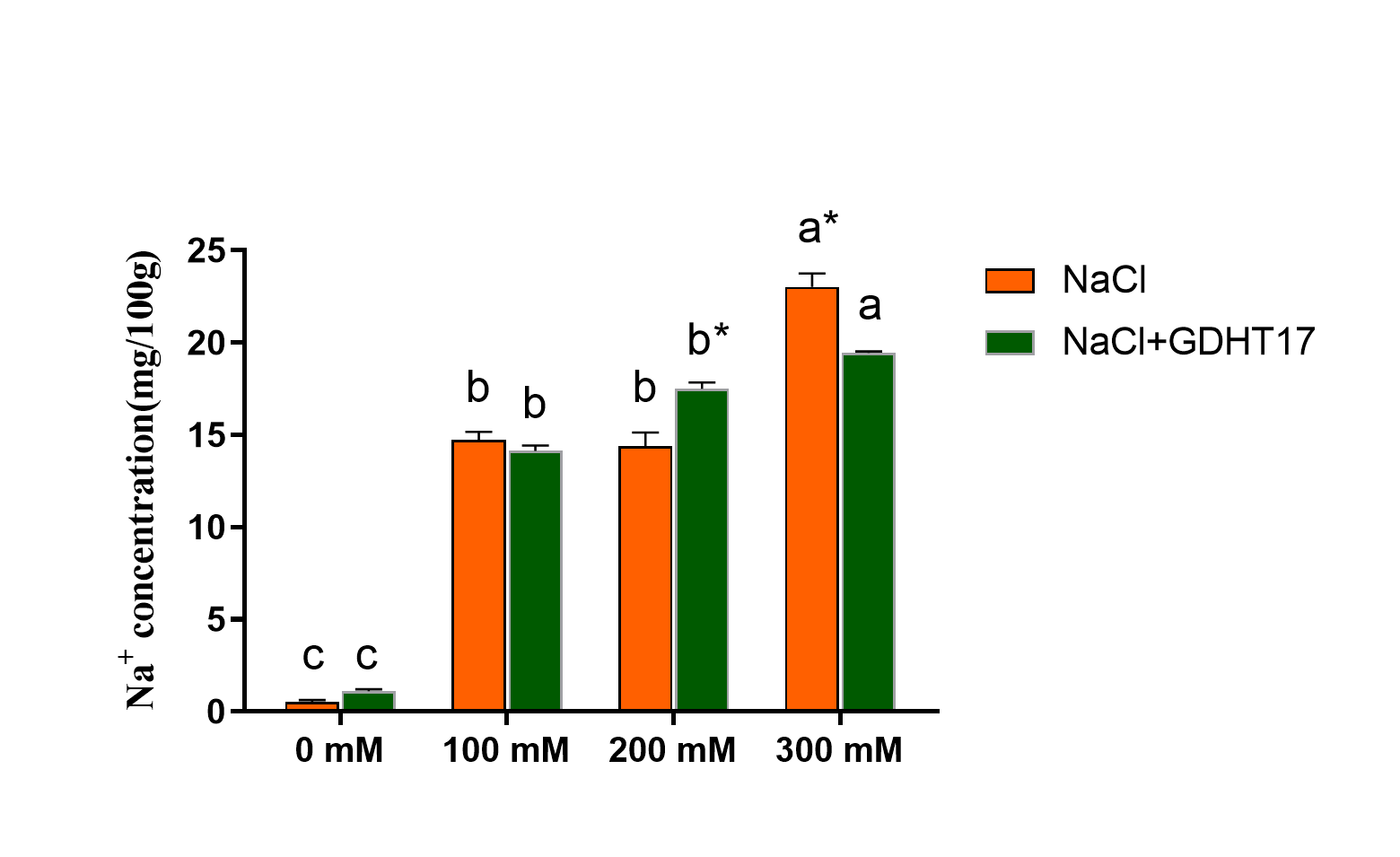

Supplement: Supplementary file 2 [file Data_Sheet_2.ZIP › Figure/Figure 7-A.tif]

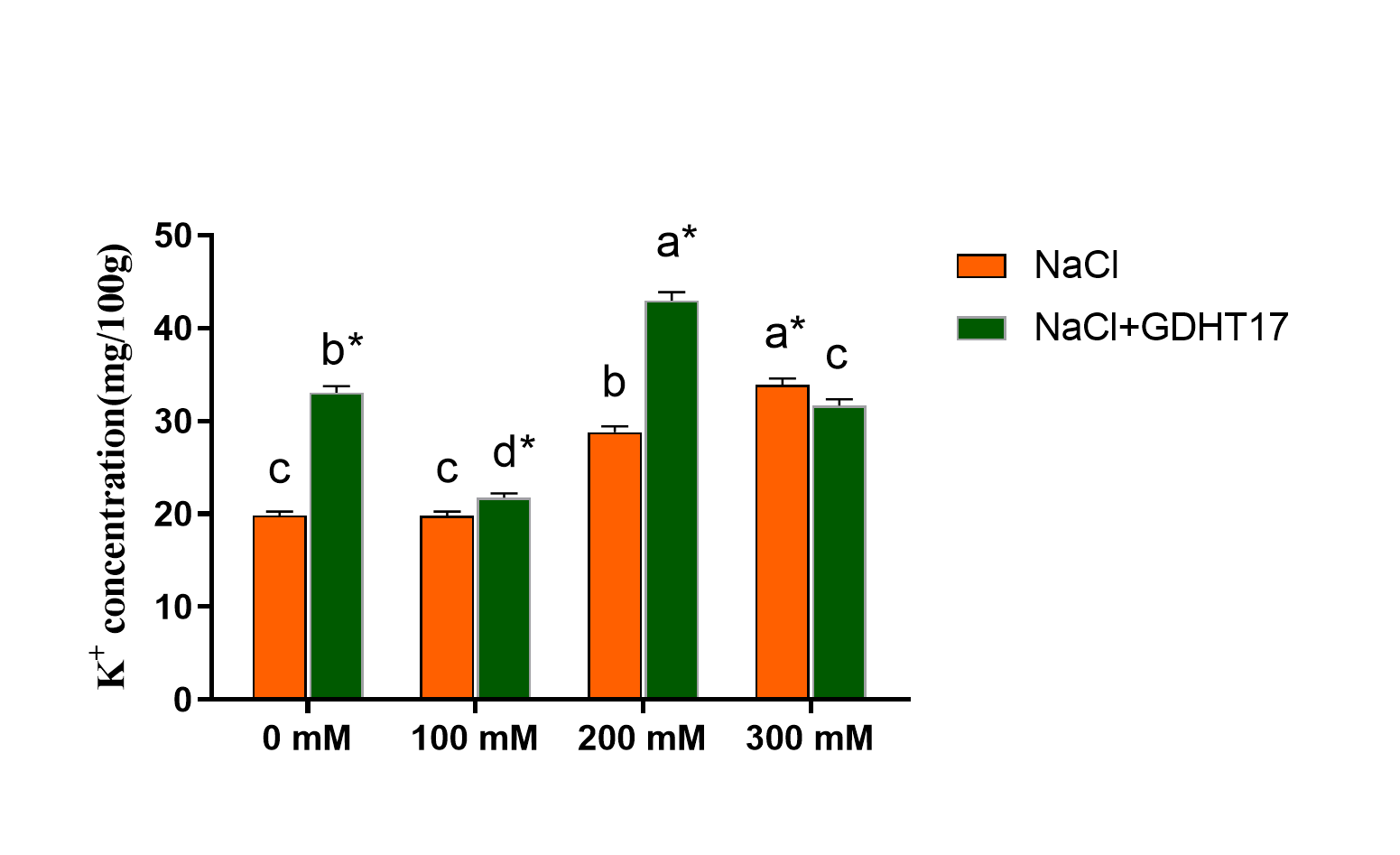

Supplement: Supplementary file 2 [file Data_Sheet_2.ZIP › Figure/Figure 7-B.tif]

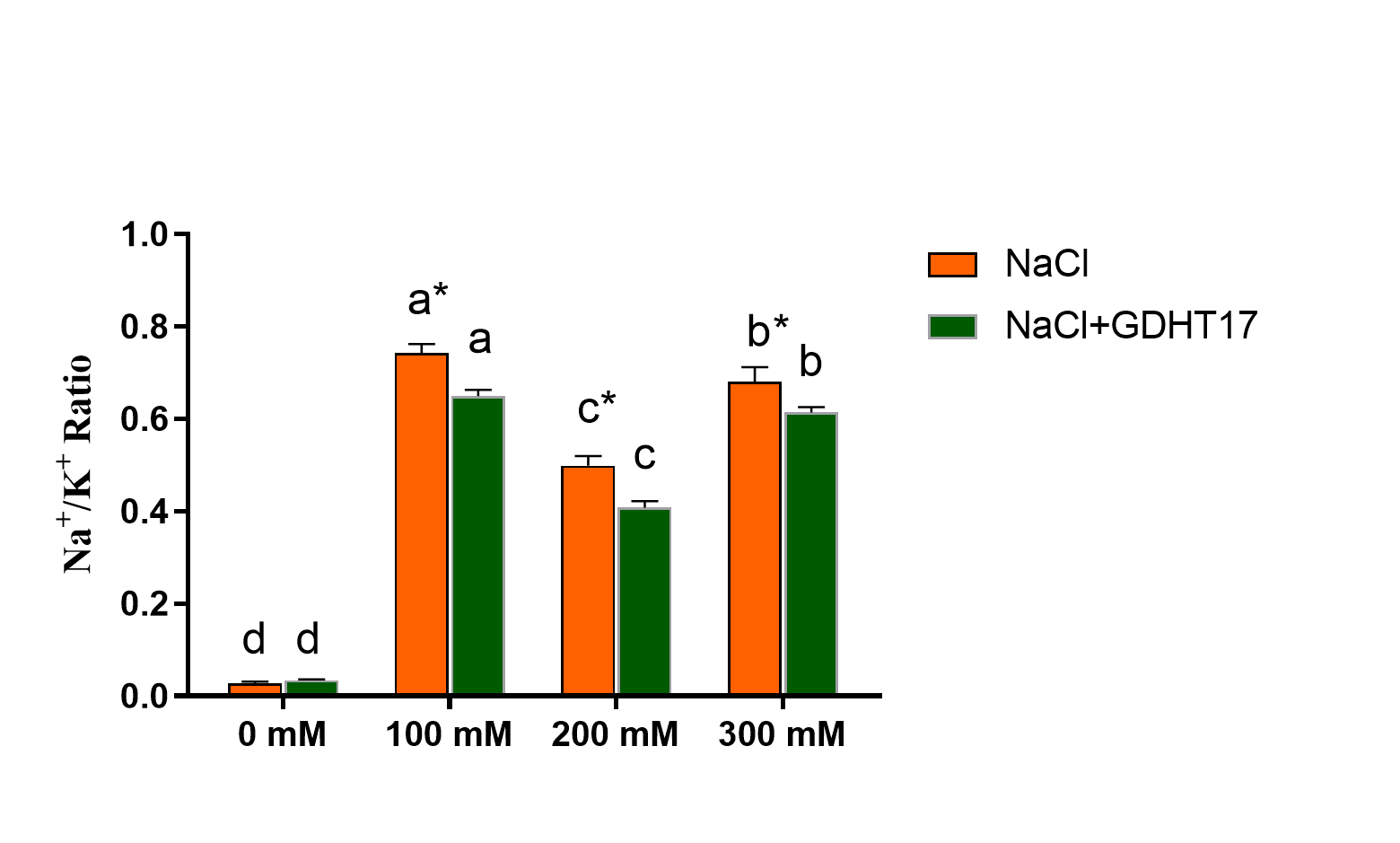

Supplement: Supplementary file 2 [file Data_Sheet_2.ZIP › Figure/Figure 7-C.tif]

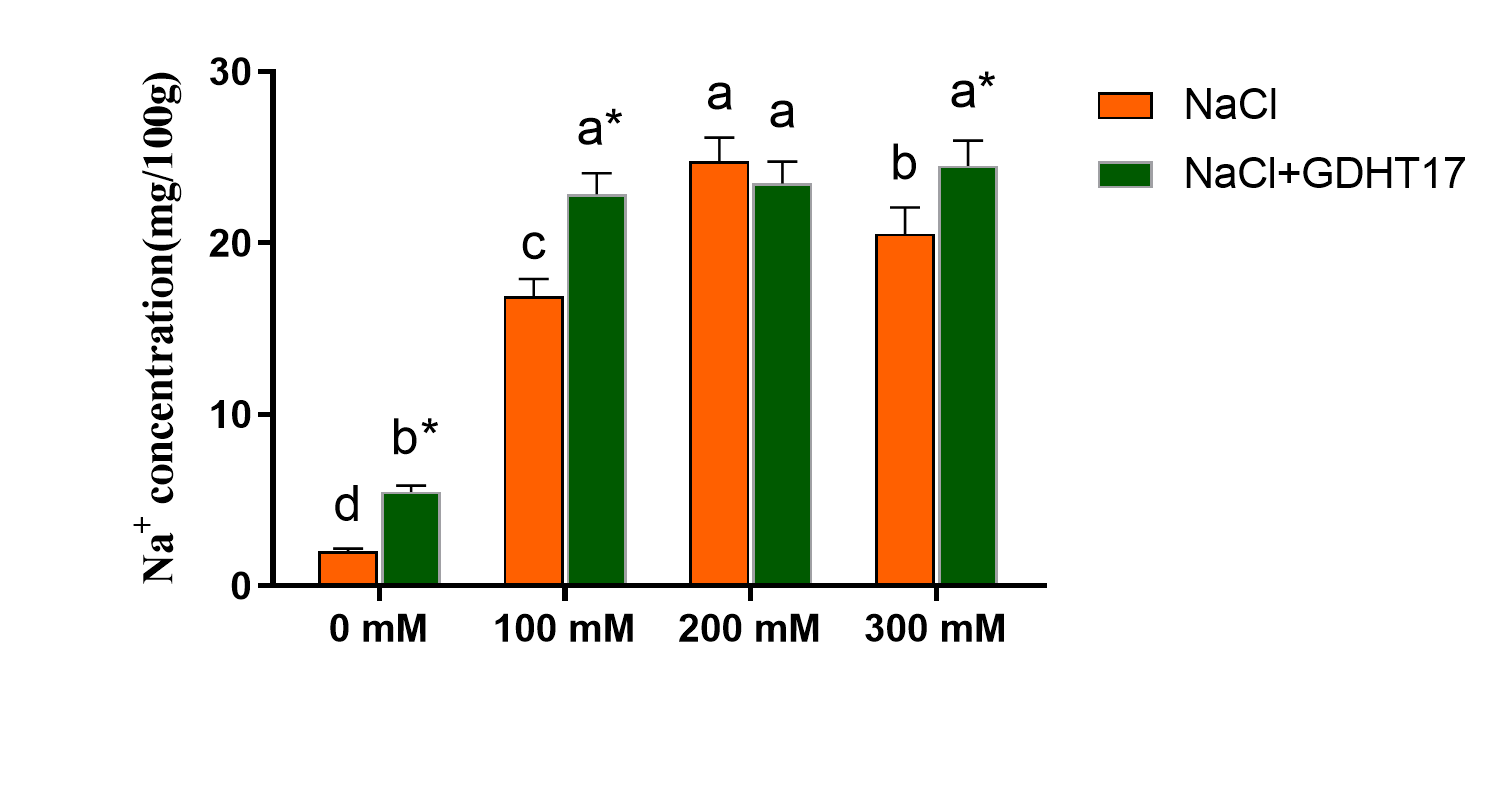

Supplement: Supplementary file 2 [file Data_Sheet_2.ZIP › Figure/Figure 7-D.tif]

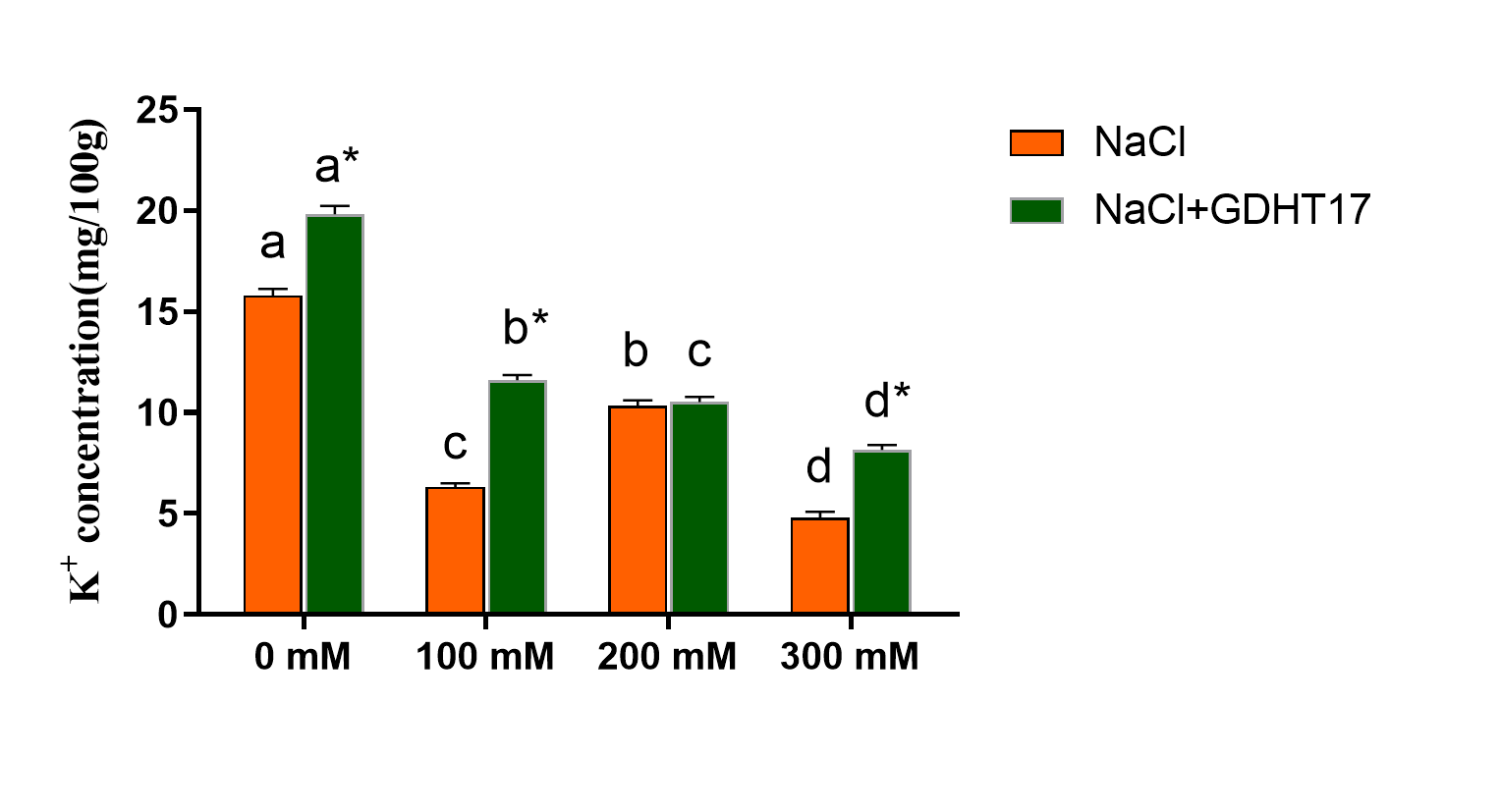

Supplement: Supplementary file 2 [file Data_Sheet_2.ZIP › Figure/Figure 7-E.tif]

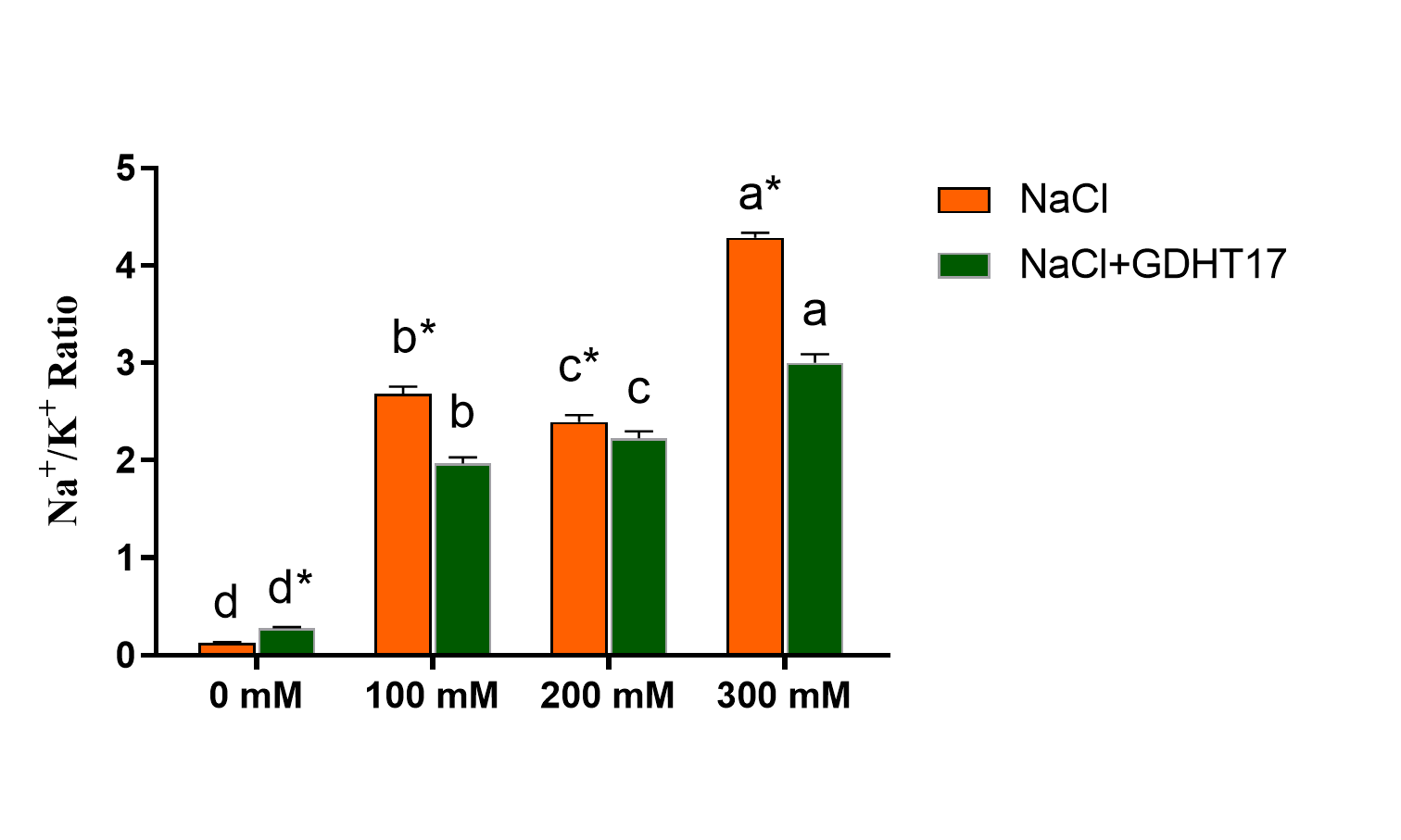

Supplement: Supplementary file 2 [file Data_Sheet_2.ZIP › Figure/Figure 7-F.tif]

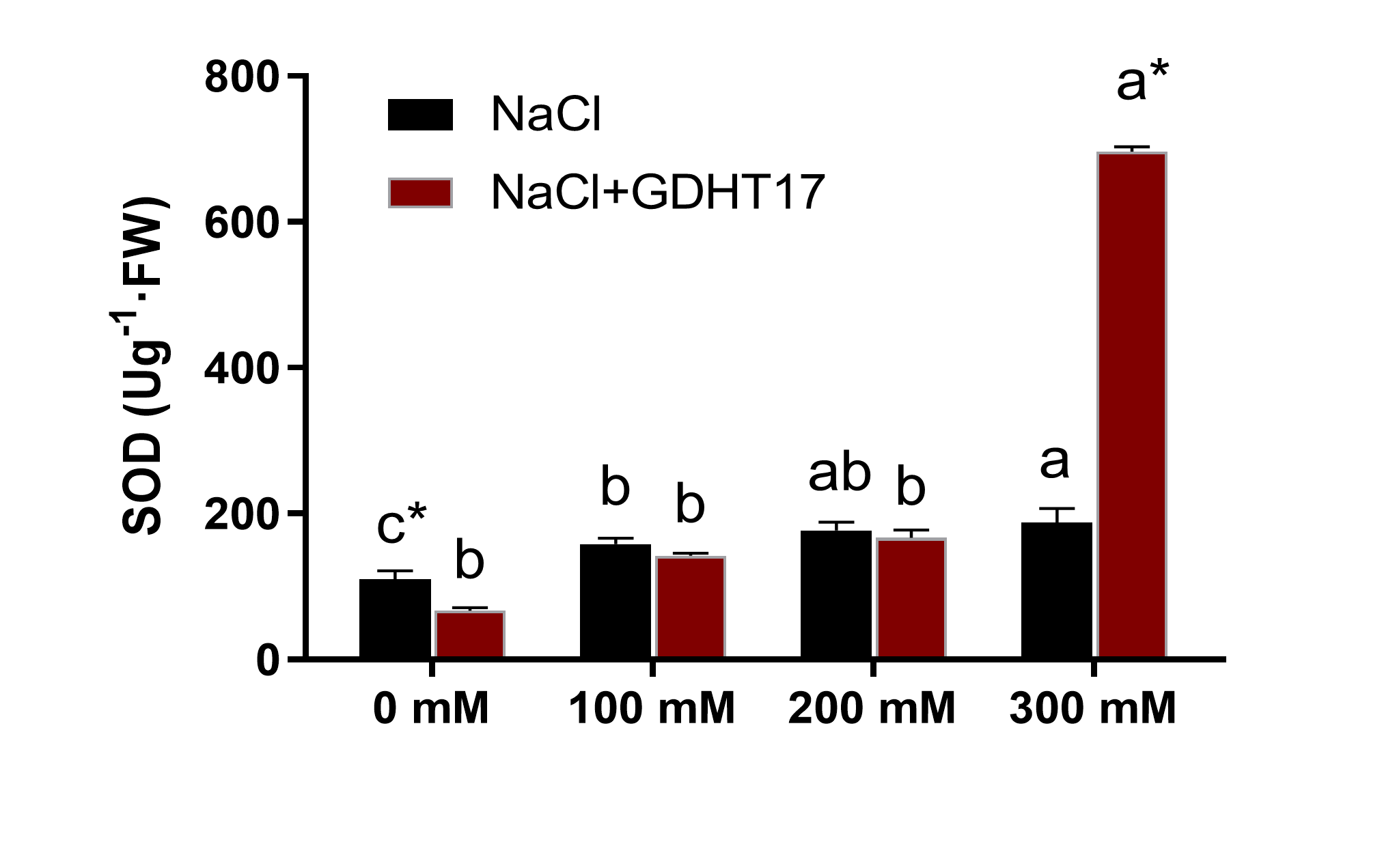

Supplement: Supplementary file 2 [file Data_Sheet_2.ZIP › Figure/Figure 8-A.tif]

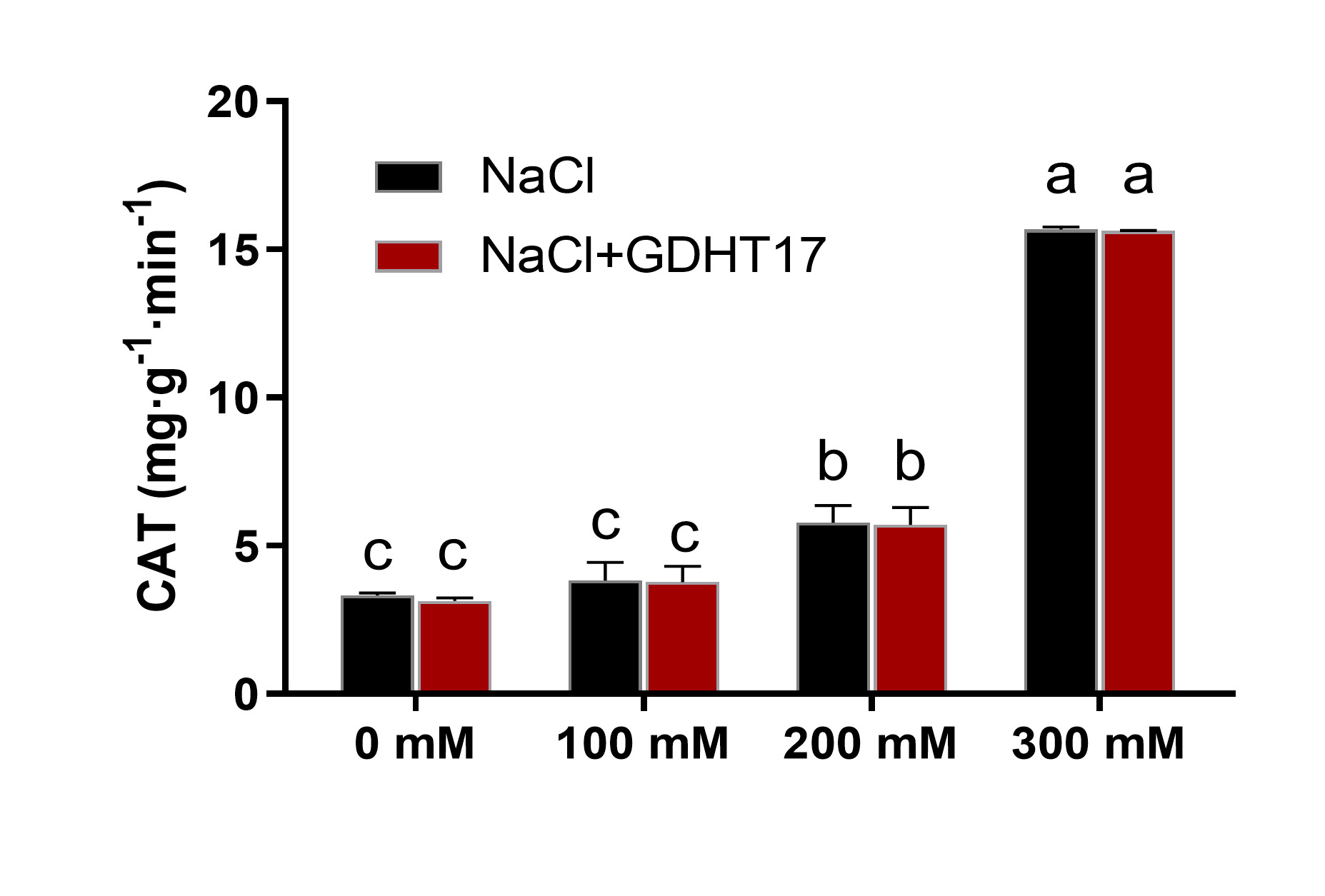

Supplement: Supplementary file 2 [file Data_Sheet_2.ZIP › Figure/Figure 8-B.tif]

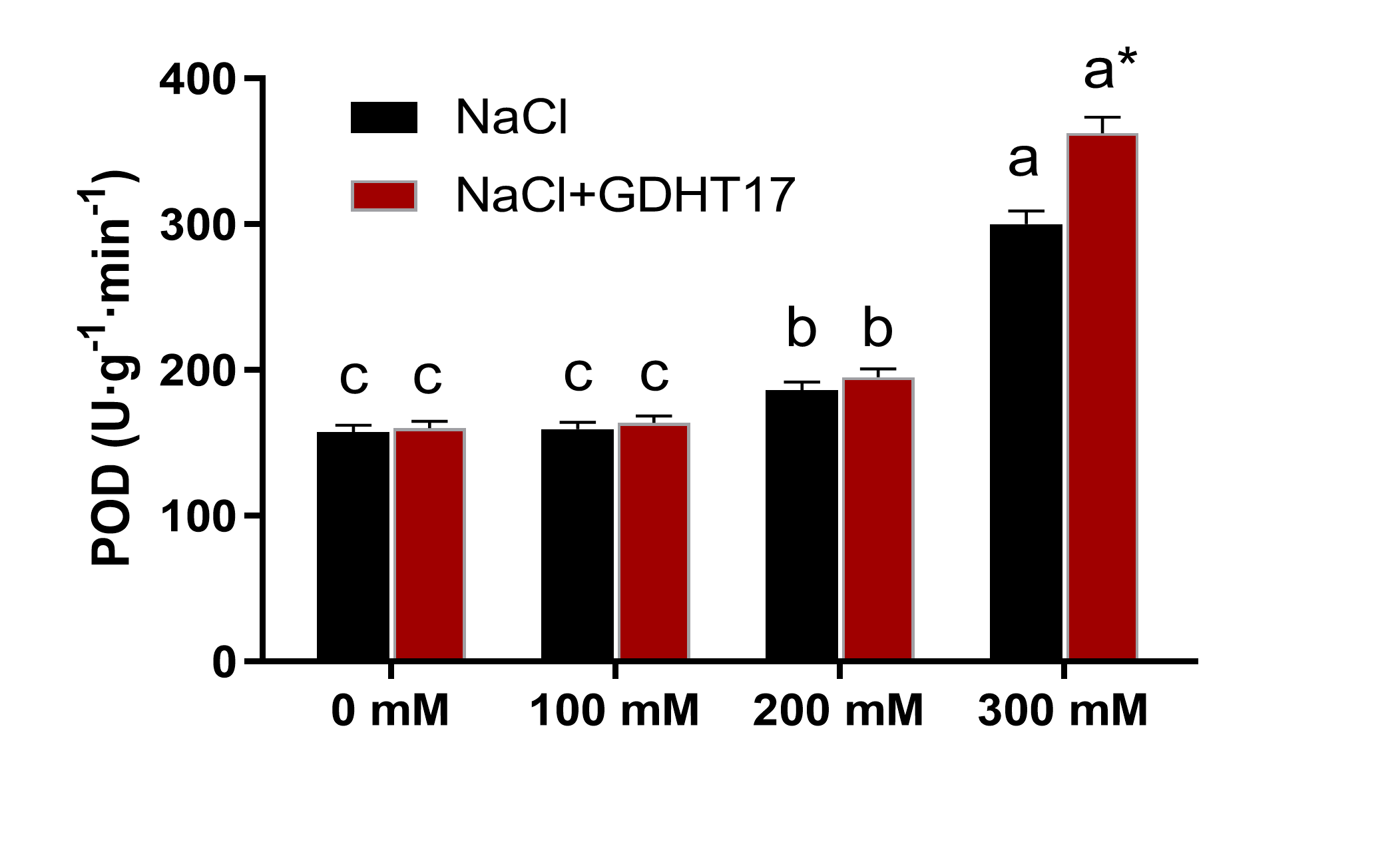

Supplement: Supplementary file 2 [file Data_Sheet_2.ZIP › Figure/Figure 8-C.tif]
